# Pregnancy-Related Disease Outcomes in Women With Moderate to Severe Multiple Sclerosis Disability

**DOI:** 10.1001/jamanetworkopen.2025.31581

**Published:** 2025-09-15

**Authors:** Jessica Shipley, Heidi N. Beadnall, Paul G. Sanfilippo, Wei Zhen Yeh, Dana Horakova, Eva Kubala Havrdova, Pavel Hradilek, Tomas Kalincik, Izanne Roos, Alexandre Prat, Marc Girard, Zuzana Rous, Zbysek Pavelek, Oliver H. H. Gerlach, Jeannette Lechner-Scott, Raed Alroughani, Serkan Ozakbas, Marek Peterka, Katherine Buzzard, Olga G. Skibina, Davide Maimone, Matteo Foschi, Andrea Surcinelli, Rana Karabudak, Daniele L. A. Spitaleri, Alessandra Lugaresi, Valentina Tomassini, Riadh Gouider, Saloua Mrabet, Beatriz Romero Ferrando, Suzanne Hodgkinson, Pavel Štourač, Joana Guimarães, Nevin A. John, Richard A. L. Macdonell, Jose E. Meca-Lallana, Helmut Butzkueven, Anneke van der Walt, Vilija G. Jokubaitis

**Affiliations:** 1 Department of Neuroscience, School of Translational Medicine, Monash University, Melbourne, Victoria, Australia; 2 Department of Neurology, Alfred Health, Melbourne, Victoria, Australia; 3 Brain and Mind Centre, The University of Sydney, Sydney, New South Wales, Australia; 4 Department of Neurology and Center of Clinical Neuroscience, First Faculty of Medicine, Charles University and General University Hospital, Prague, Czech Republic; 5 Department of Neurology, Faculty of Medicine, University of Ostrava, Ostrava, Czech Republic; 6 Neuroimmunology Centre, Department of Neurology, Royal Melbourne Hospital, Melbourne, Victoria, Australia; 7 Clinical Outcomes Research (CORe) Unit, Department of Medicine, University of Melbourne, Melbourne, Victoria, Australia; 8 Centre Hospitalier de l’Université de Montréal (CHUM) and Universite de Montreal, Montreal, Quebec, Canada; 9 Department of Neurology, Faculty of Medicine, Palacký University and University Hospital Olomouc, Olomouc, Czech Republic; 10 Department of Neurology, Faculty of Medicine and University Hospital Hradec Králové, Charles University, Prague, Czech Republic; 11 Academic MS Center Zuyd, Department of Neurology, Zuyderland Medical Center, Sittard-Geleen, Netherlands; 12 School for Mental Health and Neuroscience, Department of Neurology, Maastricht University Medical Center, Maastricht, Netherlands; 13 Hunter Medical Research Institute, University of Newcastle, Newcastle, New South Wales, Australia; 14 Hunter New England Health, John Hunter Hospital, Newcastle, New South Wales, Australia; 15 Division of Neurology, Department of Medicine, Amiri Hospital, Sharq, Kuwait; 16 Izmir University of Economics, Medical Point Hospital, Izmir, Turkey; 17 Multiple Sclerosis Research Association, Izmir, Turkey; 18 Department of Neurology, Faculty of Medicine and University Hospital in Pilsen, Charles University, Prague, Czech Republic; 19 Department of Neurology, Box Hill Hospital, Melbourne, Victoria, Australia; 20 Department of Neurosciences, Eastern Health Clinical School, Monash University, Melbourne, Victoria, Australia; 21 Centro Sclerosi Multipla, Unità Operativa Complessa di Neurologia, Azienda Ospedaliera per l’Emergenza Cannizzaro, Catania, Italy; 22 Department of Neuroscience, MS Center, Neurology Unit, Santa Maria delle Croci Hospital, Azienda Unità Sanitaria Locale (AUSL) Romagna, Ravenna, Italy; 23 Department of Biotechnological and Applied Clinical Sciences, University of L’Aquila, L’Aquila, Italy; 24 Department of Neurological Sciences, Faculty of Medicine, Yeditepe University, Istanbul, Turkey; 25 Clinical Neuroimmunology and MS Unit, Koşuyolu Hospital, Istanbul, Turkey; 26 Azienda Ospedaliera di Rilievo Nazionale San Giuseppe Moscati Avellino, Avellino, Italy; 27 Dipartimento di Scienze Biomediche e Neuromotorie, Università di Bologna, Bologna, Italia; 28 Istituto di Ricovero e Cura a Carattere Scientifico (IRCCS) Istituto delle Scienze Neurologiche di Bologna, Bologna, Italia; 29 Institute for Advanced Biomedical Technologies (ITAB), Department of Neurosciences, Imaging and Clinical Sciences, Gabriele d'Annunzio University of Chieti and Pescara, Chieti, Italy; 30 Centre for Rehabilitation, Disability and Sport Medicine (CARES), Department of Neurosciences, Imaging and Clinical Sciences, Gabriele d'Annunzio University of Chieti and Pescara, Chieti, Italy; 31 MS Centre, Clinical Neurology, Santissima Annunziata University Hospital, Chieti, Italy; 32 Department of Neurology, Clinical Investigation Centre for Neurosciences and Mental Health, Razi University Hospital, Tunis, Tunisia; 33 Faculty of Medicine of Tunis, University of Tunis El Manar, Tunis, Tunisia; 34 Department of Neurology, Waikato Hospital, Hamilton, New Zealand; 35 Immune Tolerance Laboratory Ingham Institute and Department of Medicine, University of New South Wales, Sydney, New South Wales, Australia; 36 Department of Neurology, Masaryk University Brno and University Hospital, Brno, Czech Republic; 37 Department of Neurology, Centro Hospitalar Universitario de São João, Porto, Portugal; 38 Department of Medicine, School of Clinical Sciences, Monash University, Clayton, Victoria, Australia; 39 Department of Neurology, Monash Health, Clayton, Victoria, Australia; 40 Austin Health, Melbourne, Victoria, Australia; 41 Multiple Sclerosis Centro, Servicio y Unidad de Referencia (CSUR), Clinical Neuroimmunology Unit, Neurology Department, Virgen de la Arrixaca Clinical University Hospital (IMIB-Arrixaca), Murcia, Spain; 42 Neuroinmunología Clínica y Esclerosis Múltiple (NICEM) Cathedra, Universidad Católica de Murcia (UCAM)–San Antonio Catholic University, Murcia, Spain

## Abstract

**Importance:**

Understanding the association between pregnancy and clinical outcomes in women with moderate to severe multiple sclerosis (MS) disability is crucial for guiding family planning and management strategies.

**Objective:**

To assess peripregnancy relapse activity and disability progression in women with a preconception Expanded Disability Status Scale (EDSS) score of 3 or higher.

**Design, Setting, and Participants:**

This multicenter retrospective cohort study used data from the MSBase Registry, with clinical observations spanning 1984 through 2024. Study cohorts included pregnant women with MS with a preconception EDSS score of 3 or higher (range: 3-10, with higher scores indicating more severe MS-related disability) and propensity score–matched nonpregnant women with MS (controls).

**Main Outcomes and Measures:**

The main outcomes were peripregnancy annualized relapse rates (ARRs) and time to 6-month confirmed disability worsening (CDW).

**Results:**

A total of 1631 women with MS were included, of whom 575 were in the pregnant cohort (median [IQR] age at pregnancy, 32.5 [29.1-36.1] years) and 1056 were in the nonpregnant cohort (median [IQR] age, 32.6 [27.5-37.2] years). The median (range) preconception EDSS score was 3.5 (3.0-7.5). Relapse activity decreased during pregnancy, with a 75% reduction in ARR during the first trimester (rate ratio [RR], 0.25; 95% CI, 0.15-0.43), and increased to 36% above preconception levels in the first 3 months post partum (RR, 1.36; 95% CI, 1.06-1.75). Relapse during pregnancy was associated with a higher preconception ARR (odds ratio [OR], 1.56; 95% CI, 1.10-2.20) and preconception use of natalizumab (OR, 4.42; 95% CI, 1.24-23.57) or fingolimod (OR, 14.07; 95% CI, 2.81-91.30). Older age (OR, 0.92; 95% CI, 0.85-0.99) and continuation of disease-modifying therapy into pregnancy (OR, 0.42; 95% CI, 0.19-1.00) were associated with reduced risk. Disease-modifying therapy reinitiation within 1 month post partum was associated with lower odds of early postpartum relapse (OR, 0.45; 95% CI, 0.23-0.86). There was no significant difference in time to CDW between the pregnant and nonpregnant groups (hazard ratio [HR], 1.15; 95% CI, 0.96-1.38). However, ARR during pregnancy (HR, 1.37; 95% CI, 1.13-1.65) and postpartum EDSS score higher than 4 (HR, 2.69; 95% CI, 1.80-4.03) were associated with shorter time to CDW.

**Conclusions and Relevance:**

In this cohort study, women with moderate to severe MS disability exhibited a pattern of peripregnancy relapse activity similar to that reported in women with less disability. Pregnancy was not associated with worse long-term disability outcomes, although optimizing disease control in the peripregnancy period remained critical.

## Key Points

**Question:**

How does pregnancy affect relapse activity and disability progression in women with moderate to severe multiple sclerosis–related disability?

**Findings:**

In this cohort study involving 1631 pregnant and nonpregnant women with MS, among those with live-birth pregnancies and an EDSS score of 3 or higher, relapse rates decreased by 59% to 75% during pregnancy and increased to 36% above preconception rates in the first 3 months post partum. There was no association between pregnancy history and time to disability worsening when comparing matched pregnant and nonpregnant women, but gestational relapses were associated with shorter time to disability progression.

**Meaning:**

While pregnancy history was not associated with long-term disability outcomes in this population, minimizing peripregnancy relapse activity remains essential.

## Teaser

This cohort study examines risk of relapse and disability progression in pregnant and nonpregnant women with moderate to severe multiple sclerosis–related disability.

## Introduction

Multiple sclerosis (MS) is an inflammatory demyelinating and degenerative disease of the central nervous system that commonly presents in women of reproductive age (mean age of onset, 32 years).^1^ Pregnancy planning is therefore a frequent and important aspect of MS clinical care. However, prior research on clinical outcomes associated with pregnancy has largely focused on women with minimal MS-related disability, characterized by average preconception Expanded Disability Status Scale (EDSS) scores of 0 to 2.^2,3,4,5,6,7,8,9,10,11,12,13,14,15^ As a result, there is a substantial gap in understanding how pregnancy affects women with more severe disability.

Studies in women with lower preconception disability scores from referral-center cohorts have found decreased relapse rates during pregnancy, particularly in the third trimester, followed by increased relapses in the first 3 months post partum.^2,3,4,5,6,7^ Beyond the immediate peripregnancy period, most longer-term studies have suggested that pregnancy has favorable implications^9,10,11^ or no implications for disability progression.^6,7,8,13,14,15^ However, poorer outcomes have been linked to higher preconception EDSS scores, including increased risk of peripartum relapse^4,5,6,7,16,17^ and disability worsening both during and after pregnancy.^4,15,18^ While these findings suggest heightened risks for women with more severe disability, further research is needed to improve our understanding of pregnancy-related disease outcomes in this group, enabling more informed family planning counseling and management strategies.

This study aimed to assess peripregnancy relapse activity in women with MS with a preconception EDSS score of 3 or higher, including factors associated with relapse during and after pregnancy. We also aimed to investigate the association between pregnancy history and disability worsening by comparing pregnant women and matched nonpregnant women with higher disability scores. We hypothesized that relapse patterns would mirror those observed in studies of women with lower disability and that pregnancy would not be associated with worse long-term disability outcomes.

## Methods

### Design and Data Extraction

This multicenter retrospective observational cohort study used data from the MSBase Registry,^19^ a global clinical outcomes registry that prospectively collects data on patients with neuroimmunological conditions, including MS. Data were extracted on June 2, 2024, and included all available entries from the registry’s inception to the extraction date. The MSBase Registry has ethics approval from the Alfred Health Human Research Ethics Committee and approval or exemption from the research ethics committees at each participating MSBase referral center. Written informed consent was obtained from all enrolled patients in accordance with the Declaration of Helsinki.^20^ We followed the Strengthening the Reporting of Observational Studies in Epidemiology (STROBE) reporting guideline.^21^

### Participants

Initial exclusion criteria included nonfemale sex, diagnoses other than MS, and insufficient or incongruent data (eg, missing MS symptom onset date). Women were included in the pregnant cohort if they had a pregnancy after MS onset, were aged 18 to 45 years at the time of their last menstrual period, and had an EDSS score of 3 or higher (range: 3-10, with higher scores indicating more severe MS-related disability) within 12 months before pregnancy. Only the first pregnancy meeting these criteria was included. Among these women, those with relapse-onset MS and at least 1 postconception visit were included in the relapse analysis. For the disability progression analysis, participants were required to have at least 2 postpartum visits with EDSS scores spaced at least 6 months apart.

The nonpregnant cohort comprised women without a documented pregnancy who had at least 2 consecutive EDSS scores of 3 or higher. For further screening, baseline was defined as the date of the first consecutive EDSS score of 3 or higher. Inclusion criteria required women to be aged 18 to 45 years at baseline, have at least 2 postbaseline visits with EDSS scores spaced at least 6 months apart, and have at least 3 months of prebaseline visit data to generate baseline covariates.

Propensity score matching (PSM) was performed to balance covariates between the pregnant and nonpregnant cohorts, including MS phenotype, age, disease duration, baseline EDSS score, annualized relapse rate (ARR) in the prebaseline year, and use of high-efficacy disease-modifying therapy (DMT) in the prebaseline year. High-efficacy DMT was defined as alemtuzumab, anti-CD20 therapies, natalizumab, cladribine, sphingosine-1-phosphate receptor inhibitors, cyclophosphamide, and autologous hematopoietic stem cell transplant. Covariates were considered balanced if the absolute standardized mean difference was less than 0.1. Inverse probability of treatment weighting was not used due to the presence of extreme weights. The main outcomes were peripregnancy ARRs and time to 6-month confirmed disability worsening (CDW).

### Statistical Analysis

ARRs were calculated for the 3-month intervals in the year prior to pregnancy, during pregnancy, and the year post partum, stratified by birth outcome (live birth or pregnancy loss), preconception DMT type, and treatment epoch (early: before 2005, middle: 2005-2010, or modern: after 2010). Live births included term (≥37 weeks) and preterm (24-37 weeks) births. Mixed-effects Poisson regression was used to calculate rate ratios (RRs) for relapse counts relative to the preconception year, incorporating participant-level intercepts to account for within-participant correlation and an offset term to adjust for varying observation periods. No overdispersion was observed in any model, with all dispersion parameters less than 1 (range, 0.20-0.56).

Factors potentially associated with relapse during pregnancy and the first 3 months post partum were assessed in women with live births using both univariable and multivariable logistic regression. Due to the absence of gestational relapses in individuals treated with anti-CD20 therapies, the Firth correction was applied to the preconception DMT variable. Multivariable model variables were selected a priori based on established literature regarding their association with the outcome of interest as well as the availability of a sufficient sample size.

The association between pregnancy history and disability progression, measured by 6-month CDW, was evaluated using a log-rank test and Cox proportional hazards regression model adjusted for postpartum high-efficacy DMT as a time-varying covariate. For the pregnant cohort, baseline was the preconception clinical visit date. The proportional hazards assumption was verified using both global and individual Schoenfeld residuals. Six-month CDW was defined as at least a 1.5-point increase in EDSS score from a baseline of 0.0, a 1.0-point or higher increase from 1.0 to 5.5, or a 0.5-point or higher increase from over 5.5, sustained for at least 6 months. Data from individuals without an event were censored at their most recent clinical visit. Factors associated with time to 6-month CDW were assessed in the pregnant cohort using Cox proportional hazards regression models.

Sensitivity analyses were performed to evaluate factors associated with relapse during pregnancy and the early postpartum period for live-birth pregnancies in the modern epoch. We also evaluated the association between 6-month CDW and pregnancy history restricted to live births.

Significance was defined as *P* < .05 for all analyses. All statistical analyses were conducted using R, version 4.2.2 (R Project for Statistical Computing).

## Results

### Participant Characteristics

A total of 1631 women with MS were included, of whom 575 (with 575 pregnancies) were in the pregnant cohort and 1056 were in the nonpregnant cohort (eFigure 1 in Supplement 1). The pregnant cohort comprised women from 82 centers across 21 countries with a median (IQR) age at pregnancy of 32.5 (29.1-36.1) years (eTable 1 and eFigure 2 in Supplement 1). Women in the nonpregnant cohort had a median (IQR) age of 32.6 (27.5-37.2) years. Relevant clinical visits in the pregnant group occurred between June 1993 and May 2024, with participants contributing a total of 5107 person-years follow-up from the preconception year. Additionally, 536 (93.2%) had relapsing-remitting MS, and the median (range) preconception EDSS score was 3.5 (3.0-7.5) (eFigure 3 in Supplement 1). Four hundred sixty-nine women (81.6%) used DMT in the preconception year compared with 438 (76.2%) in the postpartum year. Median washout periods and timing of postpartum reinitiation for each DMT type are provided in eTables 2 and 3 in Supplement 1.

### Peripregnancy Relapse Rates and Rate Ratios

**Figure 1.**
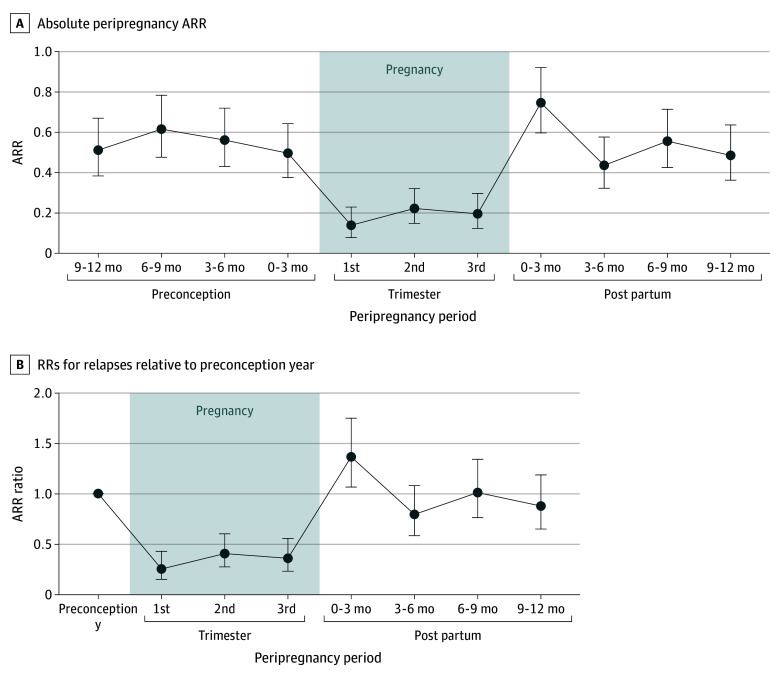
Annualized Relapse Rates (ARRs) and Rate Ratios (RRs) for Live Births RRs were calculated using a mixed-effects Poisson regression model. Error bars represent 95% CIs.

In the 469 women with live-birth pregnancies in the relapse analysis, 54 (11.5%) experienced a relapse during pregnancy and 84 (17.9%) had a relapse during the first 3 months post partum. The ARR decreased from 0.50 (95% CI, 0.38-0.64) in the preconception year to 0.14 (95% CI, 0.08-0.23) in the first trimester, 0.22 (95% CI, 0.15-0.32) in the second trimester, and 0.20 (95% CI, 0.12-0.30) in the third trimester, before increasing to 0.75 (95% CI, 0.60-0.92) in the first 3 months post partum (Figure 1A). Compared with the preconception year, there was a 75% reduction in ARR in the first trimester (RR, 0.25; 95% CI, 0.15-0.43), a 59% reduction in the second trimester (RR, 0.41; 95% CI, 0.28-0.60), a 64% reduction in the third trimester (RR, 0.36; 95% CI, 0.23-0.56), and a 36% increase in the early postpartum period (RR, 1.36; 95% CI, 1.06-1.75) (Figure 1B). The ARR then returned to baseline after 3 months post partum. For the 96 pregnancy losses, there was no significant change in ARR during pregnancy or in the early months postpregnancy loss, although the CIs were wide (eFigures 4 and 5 in Supplement 1).

**Figure 2.**
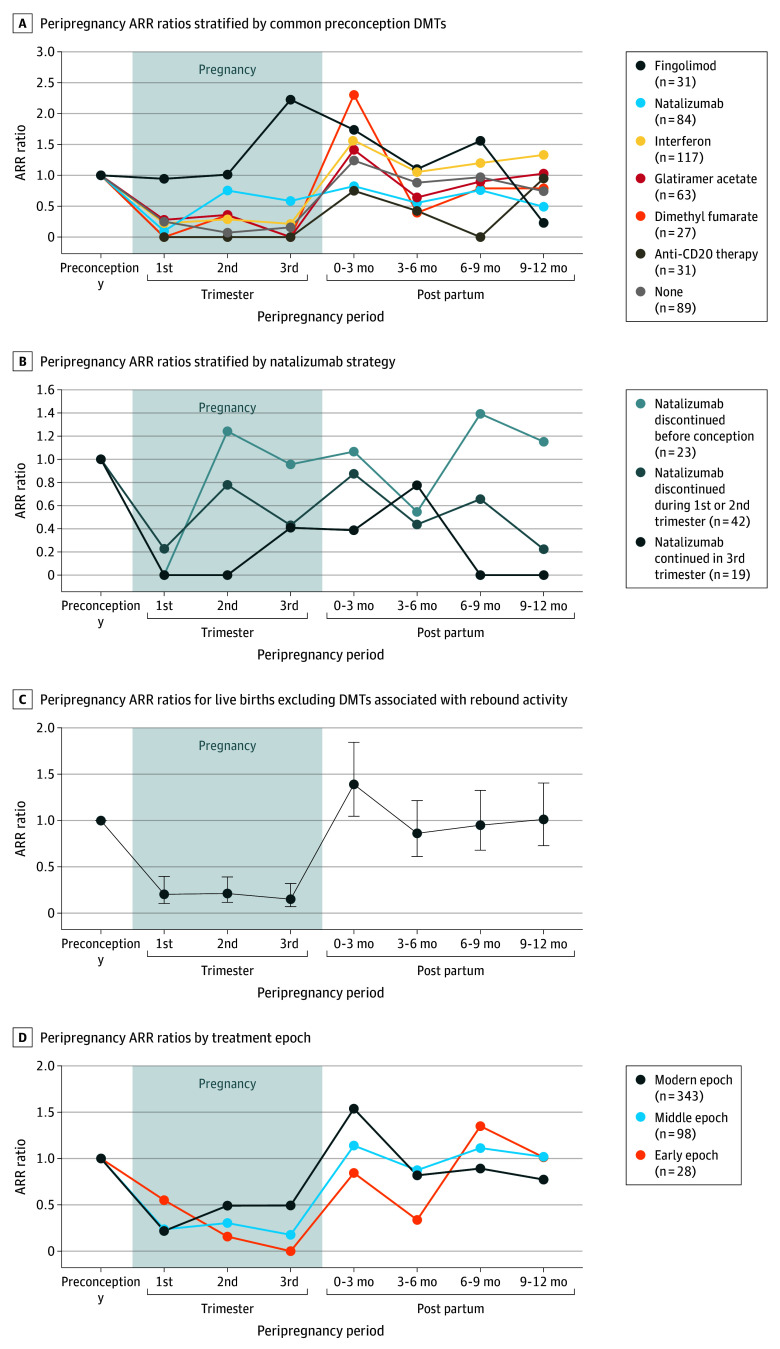
Annualized Relapse Rate (ARR) Ratios Relative to the Preconception Year by Disease-Modifying Therapy (DMT) and Treatment Epoch Rate ratios, calculated using a mixed-effects Poisson regression model, and 95% CIs for each categorical variable are detailed in eTable 4 in Supplement 1. Corresponding absolute ARR plots are included in eFigure 6 in Supplement 1. Anti-CD20 therapy included ocrelizumab (n = 25) or rituximab (n = 6). Early epoch was defined as before 2005, middle epoch as between 2005 and 2010, and modern epoch as after 2010. Error bars represent 95% CIs.

A reduction in relapse activity during pregnancy was observed in women taking most DMT types (Figure 2A; eTable 4 in Supplement 1). Statistically significant reductions occurred in the interferon (n = 117) and no treatment (n = 89) groups across all trimesters, likely due to larger sample sizes. No relapses were recorded during pregnancy in the 31 women using anti-CD20 therapies (ocrelizumab or rituximab), while the highest ARRs were seen in women using fingolimod (n = 31) or natalizumab (n = 84). In the fingolimod group, there was no reduction in relapse activity in the first 2 trimesters and a 123% increase in the third trimester (RR, 2.23; 95% CI, 1.03-4.83). Most women in this group discontinued fingolimod prior to pregnancy or during the first trimester (27 of 31 [87.1%]). While women taking natalizumab had a significant reduction in ARR in the first trimester (RR, 0.10; 95% CI, 0.01-0.71), this outcome did not persist into later stages of pregnancy. Stratification by natalizumab-prescribing strategy—discontinuation before conception (n = 23), discontinuation during the first 2 trimesters (n = 42), or continuation into the third trimester (n = 19)—suggested that the earlier discontinuation strategies likely contributed to the higher ARRs in the second and third trimesters (Figure 2B). However, small sample sizes within DMT subgroups resulted in wide CIs; therefore, these findings should be interpreted with caution (eTable 4 in Supplement 1).

Figure 2C shows the ARR ratios for live births, excluding DMT prescribing associated with rebound disease activity, specifically fingolimod use and natalizumab withdrawal before the third trimester. In this analysis, ARRs were similar across all trimesters.

Most pregnancies occurred in the modern treatment epoch (n = 343), which was associated with the lowest absolute preconception and postpartum ARRs (eFigure 6 in Supplement 1). Although relapses followed a similar pattern across treatment epochs, the traditional graded decline in relapse activity across pregnancy was not present in the middle (n = 98) and modern epochs (Figure 2D).

### Factors Associated With Relapse During Pregnancy and Early Postpartum Period

In the multivariable analysis, relapse during pregnancy was more likely with a higher preconception ARR (odds ratio [OR], 1.56; 95% CI, 1.10-2.20) and preconception use of natalizumab (OR, 4.42; 95% CI, 1.24-23.57) or fingolimod (OR, 14.07; 95% CI, 2.81-91.30). Older age at conception (OR, 0.92; 95% CI, 0.85-0.99) and continuation of DMT into pregnancy were protective against relapse (OR, 0.42; 95% CI, 0.19-1.00) (Table 1). However, these estimates—particularly for small subgroups such as fingolimod with large ORs—should be considered exploratory and interpreted with caution.

**Table 1.** Factors Associated With Relapse During Pregnancy for Live Births

Variable	No. of women with live-birth pregnancies (n = 469)	Unadjusted		Adjusted	
		OR (95% CI)	*P* value	OR (95% CI)	*P* value
Age at conception	NA	0.95 (0.89-1.01)	.08	0.92 (0.85-0.99)	.03
Disease duration at conception	NA	1.01 (0.96-1.07)	.57	1.07 (1.00-1.14)	.06
Preconception EDSS score >4	100	1.06 (0.54-2.10)	.86	1.05 (0.50-2.08)	.90
ARR in the preconception year	NA	1.54 (1.12-2.11)	.008	1.56 (1.10-2.20)	.01
DMT used within 1 mo of conception^a^^,^^b^					
Interferon	101	2.41 (0.66-12.85)	.19	2.33 (0.63-12.61)	.22
Natalizumab	78	4.33 (1.24-22.76)	.02	4.42 (1.24-23.57)	.02
Anti-CD20 therapy^c^	27	0.38 (0.00-4.91)	.50	0.31 (0.00-4.15)	.41
Dimethyl fumarate	24	1.34 (0.12-10.64)	.79	2.25 (0.19-18.68)	.47
Fingolimod	16	10.04 (2.12-61.95)	.004	14.07 (2.81-91.30)	.001
None	156	3.33 (1.02-16.96)	.05	2.02 (0.56-10.96)	.31
Monoclonal antibody therapy in the preconception year	126	1.05 (0.56-1.99)	.87	NA	NA
Natalizumab exposure relative to conception^b^					
Natalizumab discontinued before conception	23	5.21 (1.32-20.60)	.02	NA	NA
Natalizumab discontinued during 1st or 2nd trimester	42	2.95 (0.81-10.80)	.10	NA	NA
Natalizumab continued in 3rd trimester	19	0.82 (0.09-7.80)	.86	NA	NA
DMT washout period	119	1.00 (0.99-1.00)	.41	NA	NA
DMT continuation into pregnancy	235	0.60 (0.34-1.07)	.08	0.42 (0.19-1.00)	.049

Abbreviations: ARR, annualized relapse rate; DMT, disease-modifying therapy; EDSS, Expanded Disability Status Scale; NA, not applicable; OR, odds ratio.

^a^ DMT administration dates within 1 month prior to conception, or estimated infusion date within 6 months of conception for anti-CD20 therapies.

^b^ Glatiramer acetate was the reference DMT.

^c^ Includes ocrelizumab (n = 21) and rituximab (n = 6). The Firth correction was used in both the univariable and multivariable models to generate CIs for anti-CD20 therapies due to complete separation associated with the absence of relapses during pregnancy in this group.

Relapse probability increased in the first 3 months post partum in women with higher ARRs in the preconception year (OR, 1.44; 95% CI, 1.05-1.98) and decreased with DMT reinitiation within 1 month post partum (OR, 0.45; 95% CI, 0.23-0.86) (Table 2).

**Table 2.** Factors Associated With Relapse Within 3 Months Post Partum for Live Births

Variable	No. of women with live-birth pregnancies (n = 469)	Unadjusted		Adjusted	
		OR (95% CI)	*P* value	OR (95% CI)	*P* value
Age at conception	NA	0.98 (0.93-1.03)	.42	1.01 (0.94-1.07)	.87
Disease duration at conception	NA	0.97 (0.93-1.02)	.24	0.97 (0.91-1.03)	.25
Preconception EDSS score >4	100	0.84 (0.47-1.53)	.58	1.13 (0.58-2.21)	.72
ARR in the preconception year	NA	1.51 (1.15-1.99)	.003	1.44 (1.05-1.98)	.02
ARR during pregnancy	NA	1.36 (0.97-1.91)	.07	1.32 (0.91-1.92)	.15
DMT continuation into pregnancy	235	0.95 (0.63-1.44)	.81	0.99 (0.57-1.71)	.96
Natalizumab exposure relative to conception^a^					
Natalizumab discontinued before conception	23	1.00 (0.28-3.51)	.99	NA	NA
Natalizumab discontinued during 1st or 2nd trimester	42	0.36 (0.10-1.39)	.14	NA	NA
Natalizumab continued in 3rd trimester	19	0.26 (0.03-2.18)	.22	NA	NA
Breastfeeding	195	1.20 (0.75-1.93)	.45	1.16 (0.67-2.02)	.60
DMT reinitiation within 1 mo post partum	123	0.46 (0.25-0.84)	.01	0.45 (0.23-0.86)	.02
Monoclonal antibody therapy reinitiated within 1 mo post partum	57	0.41 (0.16-1.05)	.06	NA	NA
Timing of natalizumab reinitiation^b^					
Early: ≤14 d post partum	29	0.40 (0.08-2.02)	.27	NA	NA
Late: >14 d post partum	52	1.28 (0.46-3.56)	.64	NA	NA

Abbreviations: ARR, annualized relapse rate; DMT, disease-modifying therapy; EDSS, Expanded Disability Status Scale; NA, not applicable; OR, odds ratio.

^a^ Glatiramer acetate was the reference DMT.

^b^ Postpartum glatiramer acetate use was the reference DMT.

### CDW by Pregnancy History and Factors Associated With Time to CDW

**Figure 3.**
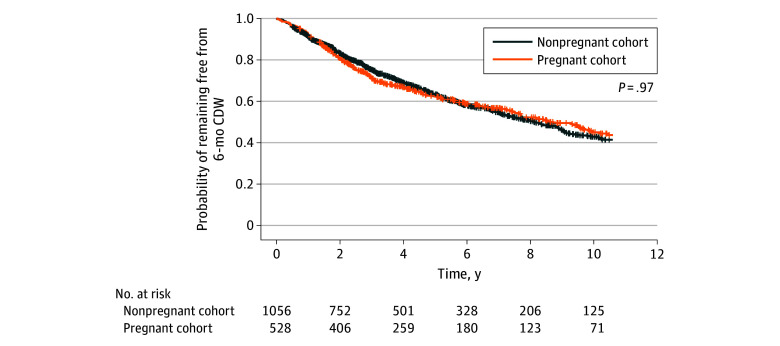
Kaplan-Meier Survival Curves by Pregnancy History CDW indicates confirmed disability worsening.

The association between pregnancy history and time to 6-month CDW was evaluated in 528 pregnant women and 1056 matched nonpregnant women with EDSS scores of 3 or higher, with successful balance between groups achieved for all covariates using 2:1 PSM (eTable 5 and eFigure 7 in Supplement 1). Relevant clinical visits in the nonpregnant group spanned August 1984 to May 2024. Kaplan-Meier curves demonstrated significant overlap for at least 10 years after baseline, with the log-rank test indicating no difference in CDW rates between the groups (*P* = .97) (Figure 3). Additionally, after adjusting for high-efficacy DMT as a time-varying covariate, there was no significant difference in time to CDW between the pregnant and nonpregnant women (hazard ratio [HR], 1.15; 95% CI, 0.96-1.38) (eTable 6 in Supplement 1). The proportional hazards assumption was satisfied, supporting the validity of the Cox proportional hazards regression model (eTable 7 and eFigure 8 in Supplement 1). For women with live-birth pregnancies, ARR during pregnancy (HR, 1.37; 95% CI, 1.13-1.65) and postpartum EDSS score higher than 4 (HR, 2.69; 95% CI, 1.80-4.03) were independently associated with shorter time to 6-month CDW (eTable 8 in Supplement 1).

### Sensitivity Analyses

Sensitivity analyses, which were restricted to women with live births in the modern epoch, identified similar factors associated with gestational and postpartum relapses (eTables 9-10 in Supplement 1). However, preconception ARR was not statistically significant for postpartum relapse in the multivariable analysis. A sensitivity analysis of time to 6-month CDW in women with live-birth pregnancies was consistent with the main analysis of women with pregnancies of any duration (eTable 11 in Supplement 1).

## Discussion

Overall, our study cohort of women from MSBase referral centers demonstrated a pattern of relapse activity during and after pregnancy similar to that reported in women with minimal disability^2,3,4,6,7,8,22^ as well as a smaller cohort of women with higher EDSS scores.^18^ During pregnancy, ARRs decreased by 59% to 75%, followed by disease reactivation in the early postpartum period with a 36% higher relapse rate in the first 3 months post partum compared with the preconception year. After excluding women at risk of drug-induced rebound activity (those using fingolimod or who discontinued natalizumab before the third trimester^2,23^), ARRs further decreased during pregnancy, with similar rates across all trimesters.

Absolute ARRs in the peripregnancy period were also comparable to ARRs reported in a 2011 meta-analysis of 22 studies involving 13 144 women with MS.^24^ In our cohort, ARRs across live-birth pregnancies were 0.50 (95% CI, 0.38-0.64) in the prepregnancy year, 0.14 (95% CI, 0.08-0.23) in the first trimester, and 0.75 (95% CI, 0.60-0.92) in the first 3 months post partum. In comparison, the meta-analysis reported ARRs of 0.44 (95% CI, 0.40-0.72) for prepregnancy, 0.26 (95% CI, 0.19-0.32) during pregnancy, and 0.76 (95% CI, 0.64-0.87) after delivery.^24^ Furthermore, women in our study with pregnancies in the modern MS treatment epoch had peripartum ARRs similar to those observed in a contemporary cohort of women with lower disability scores (median [IQR] EDSS score, 1.5 [0.0-2.0]) in a recent study.^2^

Factors associated with an increased risk of relapse during pregnancy in the present cohort included younger age, higher ARR in the preconception year, preconception use of natalizumab or fingolimod, and discontinuation of DMT prior to pregnancy, similar to findings in women with lower disability scores.^2,4,8,11,17,18^ Preconception fingolimod use had the highest OR, with a 14-fold increased risk of relapse. Together with the known teratogenic properties of fingolimod,^25^ this substantial risk of rebound relapse reinforces that fingolimod should be avoided in women of childbearing age with higher disability scores.^2,4,12,23,26^ Furthermore, preconception withdrawal of natalizumab increased the odds of relapse during pregnancy, while continuation into the third trimester reduced the odds, although the latter did not reach statistical significance. This finding supports that continuing natalizumab into later pregnancy reduces rebound relapse risk, consistent with results in prior studies.^2,4,12^ Additionally, the absence of relapses during pregnancy in women treated with anti-CD20 therapies (ocrelizumab or rituximab) aligns with recent research.^27,28^ While continuing any DMT into pregnancy reduced relapse risk, the safety data for specific DMTs must be carefully considered.^29^

Higher preconception ARR was associated with an increased risk of relapse both during pregnancy and early post partum, highlighting the importance of optimizing disease control before pregnancy.^4,5,6,7,16^ Moreover, early reinitiation of DMT within the first month post partum significantly reduced the odds of relapse. This finding adds support to early postpartum DMT recommencement when relapse risk is heightened.^2,29,30^ While the timing of DMT recommencement needs to be balanced with the safety of each DMT during breastfeeding, increasing evidence supports the safety of contemporary monoclonal antibody therapies, including anti-CD20 therapies and natalizumab during breastfeeding, particularly after the first 2 weeks post partum.^31,32^ Overall, the findings from these analyses align with recently published clinical guidelines based on studies in women with lower disability scores, which emphasize aiming for a period of disease stability prior to pregnancy, proactive planning of DMT use around pregnancy, and early postpartum DMT reinitiation.^33,34,35^

To our knowledge, this study is the first to explore the association between pregnancy history and MS disability progression in women with higher disability scores, comparing CDW outcomes between matched pregnant and nonpregnant women. Pregnancy history was not associated with time to CDW, offering reassurance for women with higher disability levels considering pregnancy. However, relapses during pregnancy were associated with shorter time to subsequent disability progression.^2,16^ Moreover, higher EDSS scores (>4) after pregnancy were associated with CDW. A higher postpartum disability score was associated with the highest adjusted HR, underscoring the importance of minimizing disability accrual during and after pregnancy. Higher disability scores early in the disease course are a well-established factor associated with long-term disability, and our findings align with this evidence base.^36^

### Limitations

While, to our knowledge, this study is the largest to examine disease outcomes in women with moderate to severe MS-related disability, sample sizes were limited for some analyses, such as stratification by DMT. Retrospective registry data also have inherent limitations, including incomplete reporting of clinical information such as pregnancy history. Although PSM balanced measured covariates between the pregnant and nonpregnant groups, matching by baseline calendar year and country was limited by sample size. Changes in MS diagnostic criteria and DMT prescribing over the study period may have introduced cohort differences. Residual imbalances from unmeasured confounders, such as social deprivation and comorbid conditions, also remain possible. Additionally, although interrater and intrarater variability in EDSS scoring and non–MS-related changes may affect the reliability of EDSS scores, a strength of this study is that we used sustained CDW.^37^ Finally, given that participants were from referral centers, findings may not be generalizable to community-based populations. Notably, a population-based study found no increased risk of postpartum relapses in women with MS.^17^ Further research is needed to confirm the study findings, particularly aimed at strategies to reduce peripartum relapse risk and disability progression in this already vulnerable group with higher disability scores.

## Conclusions

In this cohort study, women with moderate to severe MS disability exhibited a pattern of relapse activity around pregnancy similar to that reported in women with minimal disability. To improve outcomes, fingolimod should be avoided in women during childbearing years due to the significant risk of rebound relapse following withdrawal. The duration of natalizumab interruption should also be minimized. Reassuringly, pregnancy was not associated with worse long-term disability outcomes in this cohort, although optimizing disease control in the pregnancy period remained critical.

## Notes

Supplement 1. **eFigure 1.** Inclusion and Exclusion Flow Diagram **eTable 1.** Characteristics of Women in the Pregnant Cohort **eFigure 2.** Countries of Women in the Pregnant Cohort **eFigure 3.** Preconception EDSS Scores of Women in the Pregnant Cohort **eTable 2.** Washout Periods of Preconception Disease-Modifying Therapies **eTable 3.** Timing of Reinitiation of Disease-Modifying Therapies After Pregnancy **eFigure 4.** Annualised Relapse Rates and Rate Ratios for First-Trimester Pregnancy Losses **eFigure 5.** Annualised Relapse Rates and Rate Ratios for First-Trimester and Second-Trimester Pregnancy Losses **eTable 4.** Annualised Relapse Rate Ratios for Live Births by Disease-Modifying Therapy and Treatment Epoch **eFigure 6.** Annualised Relapse Rates for Live Births by Disease-Modifying Therapy and Treatment Epoch **eTable 5.** Covariate Balance Between Pregnant and Non-Pregnant Cohorts Before and After 2:1 Propensity Score Matching **eFigure 7.** Covariate Balance Between Pregnant and Non-Pregnant Cohorts Before and After 2:1 Propensity Score Matching **eTable 6.** Survival Analysis of Time to 6-Month Confirmed Disability Worsening for All Pregnancies **eTable 7.** Assessment of Proportional Hazards Assumption Using Schoenfeld Residuals **eFigure 8.** Assessment of Proportional Hazards Assumption Using Scaled Schoenfeld Residual Plots **eTable 8.** Predictors of Time to 6-Month Confirmed Disability Worsening for Pregnant Women with Live Births **eTable 9.** Predictors of Relapse During Pregnancy for Live Births in the Modern Epoch **eTable 10.** Predictors of Relapse Within 6 Months Postpartum for Live Births in the Modern Epoch **eTable 11.** Survival Analysis of Time to 6-Month Confirmed Disability Worsening for Live Births Only

Supplement 2.
Nonauthor Collaborators

Supplement 3.
Data Sharing Statement
