# Supplementary material for: Pregnancy-Related Disease Outcomes in Women With Moderate to Severe Multiple Sclerosis Disability
Source: JAMA Netw Open. 2025 Sep 15;8(9):e2531581. doi: 10.1001/jamanetworkopen.2025.31581 (PMC12439057; doi:10.1001/jamanetworkopen.2025.31581)
Supplement: Supplement 1. — eFigure 1. Inclusion and Exclusion Flow Diagram eTable 1. Characteristics of Women in the Pregnant Cohort eFigure 2. Countries of Women in the Pregnant Cohort eFigure 3. Preconception EDSS Scores of Women in the Pregnant Cohort eTable 2. Washout Periods of Preconception Disease-Modifying Therapies eTable 3. Timing of Reinitiation of Disease-Modifying Therapies After Pregnancy eFigure 4. Annualised Relapse Rates and Rate Ratios for First-Trimester Pregnancy Losses eFigure 5. Annualised Relapse Rates and Rate Ratios for First-Trimester and Second-Trimester Pregnancy Losses eTable 4. Annualised Relapse Rate Ratios for Live Births by Disease-Modifying Therapy and Treatment Epoch eFigure 6. Annualised Relapse Rates for Live Births by Disease-Modifying Therapy and Treatment Epoch eTable 5. Covariate Balance Between Pregnant and Non-Pregnant Cohorts Before and After 2:1 Propensity Score Matching eFigure 7. Covariate Balance Between Pregnant and Non-Pregnant Cohorts Before and After 2:1 Propensity Score Matching eTable 6. Survival Analysis of Time to 6-Month Confirmed Disability Worsening for All Pregnancies eTable 7. Assessment of Proportional Hazards Assumption Using Schoenfeld Residuals eFigure 8. Assessment of Proportional Hazards Assumption Using Scaled Schoenfeld Residual Plots eTable 8. Predictors of Time to 6-Month Confirmed Disability Worsening for Pregnant Women with Live Births eTable 9. Predictors of Relapse During Pregnancy for Live Births in the Modern Epoch eTable 10. Predictors of Relapse Within 6 Months Postpartum for Live Births in the Modern Epoch eTable 11. Survival Analysis of Time to 6-Month Confirmed Disability Worsening for Live Births Only [file jamanetwopen-e2531581-s001.pdf]

Supplemental Online Content

Shipley J, Beadnall HN, Sanfilippo PG, et al; the MSBase Investigator Group. Pregnancy-related disease outcomes in women with moderate to severe multiple sclerosis disability. *JAMA Netw Open*. 2025;8(9):e2531581. doi:10.1001/jamanetworkopen.2025.31581

eFigure 1. Inclusion and Exclusion Flow Diagram

eTable 1. Characteristics of Women in the Pregnant Cohort

eFigure 2. Countries of Women in the Pregnant Cohort

eFigure 3. Preconception EDSS Scores of Women in the Pregnant Cohort

eTable 2. Washout Periods of Preconception Disease-Modifying Therapies

eTable 3. Timing of Reinitiation of Disease-Modifying Therapies After Pregnancy

eFigure 4. Annualised Relapse Rates and Rate Ratios for First-Trimester Pregnancy Losses

eFigure 5. Annualised Relapse Rates and Rate Ratios for First-Trimester and Second-Trimester Pregnancy Losses

eTable 4. Annualised Relapse Rate Ratios for Live Births by Disease-Modifying Therapy and Treatment Epoch

eFigure 6. Annualised Relapse Rates for Live Births by Disease-Modifying Therapy and Treatment Epoch

eTable 5. Covariate Balance Between Pregnant and Non-Pregnant Cohorts Before and After 2:1 Propensity Score Matching

eFigure 7. Covariate Balance Between Pregnant and Non-Pregnant Cohorts Before and After 2:1 Propensity Score Matching

© 2025 Shipley J et al. *JAMA Network Open*

eTable 6. Survival Analysis of Time to 6-Month Confirmed Disability Worsening for All Pregnancies

eTable 7. Assessment of Proportional Hazards Assumption Using Schoenfeld Residuals

eFigure 8. Assessment of Proportional Hazards Assumption Using Scaled Schoenfeld Residual Plots

eTable 8. Predictors of Time to 6-Month Confirmed Disability Worsening for Pregnant Women with Live Births

eTable 9. Predictors of Relapse During Pregnancy for Live Births in the Modern Epoch

eTable 10. Predictors of Relapse Within 6 Months Postpartum for Live Births in the Modern Epoch

eTable 11. Survival Analysis of Time to 6-Month Confirmed Disability Worsening for Live Births Only

This supplemental material has been provided by the authors to give readers additional information about their work.

eFigure 1. Inclusion and Exclusion Flow Diagram

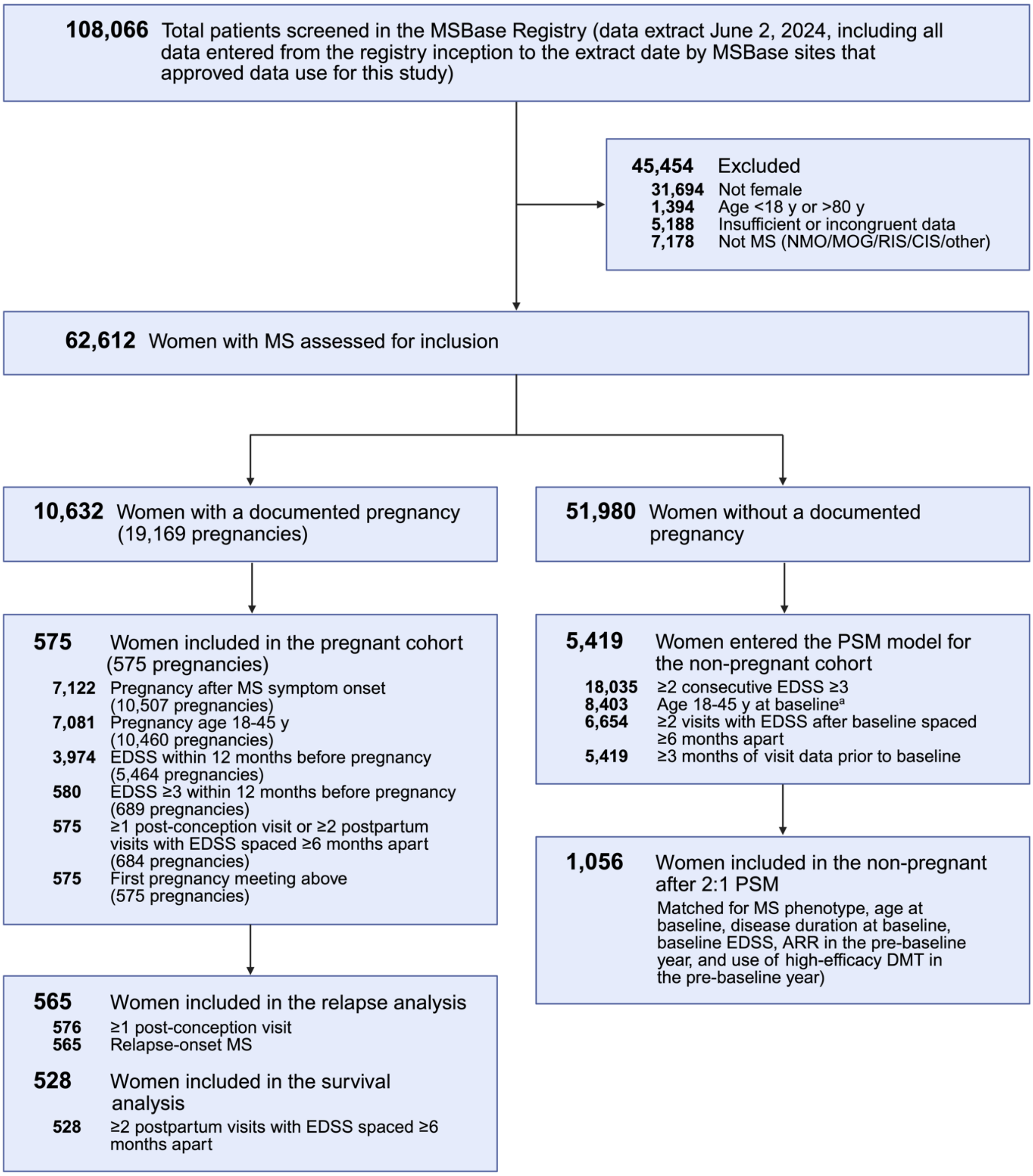

Abbreviations: ARR, annualised relapse rate; CIS, clinically isolated syndrome; DMT, disease-modifying therapy; EDSS, Expanded Disability Status Scale; MOG, myelin-oligodendrocyte glycoprotein antibody-associated disease; NMO, neuromyelitis optica spectrum disorder; PSM, propensity score matching; RIS, radiologically isolated syndrome.

[a] Baseline in the non-pregnant group was defined as the first consecutive EDSS score of at least 3

© 2025 Shipley J et al. *JAMA Network Open*

eTable 1. Characteristics of Women in the Pregnant Cohort

Characteristic	n = 575
**Age at MS symptom onset, median (IQR), y**	23.33 (19.50-27.14)
**MS phenotype, n (%)**	
**Relapsing-remitting MS**	536 (93.2)
**Secondary progressive MS**	29 (5.0)
**Primary progressive MS**	10 (1.7)
**Age at included pregnancy, median (IQR), y**	32.50 (29.14-36.10)
**Disease duration at included pregnancy, median (IQR), y**	8.17 (4.50-12.36)
**Pregnancy duration, median (IQR), w**	38.57 (34.79-39.86)
**Live births, n (%)**	477 (82.96)
**Days between preconception visit and conception, median (IQR)**	73 (30.5-140.5)
**Preconception EDSS, median (range)**	3.5 (3.0-7.5)
**ARR in the preconception year, median (IQR)**	0.00 (0.00-1.00)
**DMTs used in the preconception year, n (%)[a]**	469 (81.6)
**Interferon**	141 (24.5)
**Natalizumab**	104 (18.1)
**Glatiramer acetate**	76 (13.2)
**Fingolimod**	46 (8.0)
**Anti-CD20 therapy**	40 (7.0)[b]
**Dimethyl fumarate**	31 (5.4)
**Alemtuzumab**	9 (1.6)
**Teriflunomide**	8 (1.4)
**Cladribine**	4 (0.7)
**Other**	10 (1.7)[c]
**Women on high-efficacy DMT in the preconception year, n (%)**	205 (25.7)
**Months between DMT withdrawal and pregnancy, median (IQR)[d]**	-0.99 (-1.77-0.39)
**DMT used in the postpartum year, n (%)[e]**	438 (76.2)
**Interferon**	131 (22.8)
**Natalizumab**	103 (17.9)
**Glatiramer acetate**	62 (10.8)
**S1P receptor modulator**	50 (8.7)[f]
**Anti-CD20 therapy**	36 (6.3)[g]
**Dimethyl fumarate**	31 (5.4)
**Alemtuzumab**	9 (1.6)
**Teriflunomide**	8 (1.4)
**Cladribine**	4 (0.7)
**Other**	4 (0.7)[h]
**Women on high-efficacy DMT in the postpartum year, n (%)**	201 (35.0)
**Months to reinitiation of DMT postpartum, median (IQR)[i]**	1.46 (0.00-3.90)
**Documentation of breastfeeding, n (%)**	195 (33.9)
**Breastfeeding duration, median (IQR), mo**	2.46 (1.12-5.03)
**Follow-up duration from the preconception visit, median (IQR), y**	13.09 (8.20-7.67)

Abbreviations: DMT, disease-modifying therapy; IQR, interquartile range; mo, months; S1P, sphingosine-1-phosphate; w, weeks; y, years.

[a] For individuals who used more than one DMT type in the preconception year, only the DMT commenced closest to conception was included

[b] Ocrelizumab (n = 33), rituximab (n = 7)

[c] Mitoxantrone (n = 3), trial (n = 3), AHSCT (n = 2), daclizumab (n = 2)

[d] Positive values indicate withdrawal of the DMT prior to pregnancy, and negative values indicate continuation after conception. In some cases, a DMT may have been withdrawn for pregnancy, but the temporary discontinuation and reinitiation dates may not have been entered in the registry, which may influence the accuracy of the data

[e] For individuals who used more than one DMT type in the postpartum year, only the first DMT initiated after pregnancy was included

[f] Fingolimod (n = 48), ozanimod (n = 1), siponimod (n = 1)

[g] Ocrelizumab (n = 29), rituximab (n = 6), ofatumumab (n = 1)

[h] Mitoxantrone (n = 2), cyclophosphamide (n = 1), trial (n = 1)

[i] For DMT reinitiated within 12 months postpartum. The reinitiation time was set to 0 if a DMT was continued during pregnancy without an interval off treatment, or if it was recommenced prior to the end date of pregnancy

© 2025 Shipley J et al. *JAMA Network Open*

eFigure 2. Countries of Women in the Pregnant Cohort

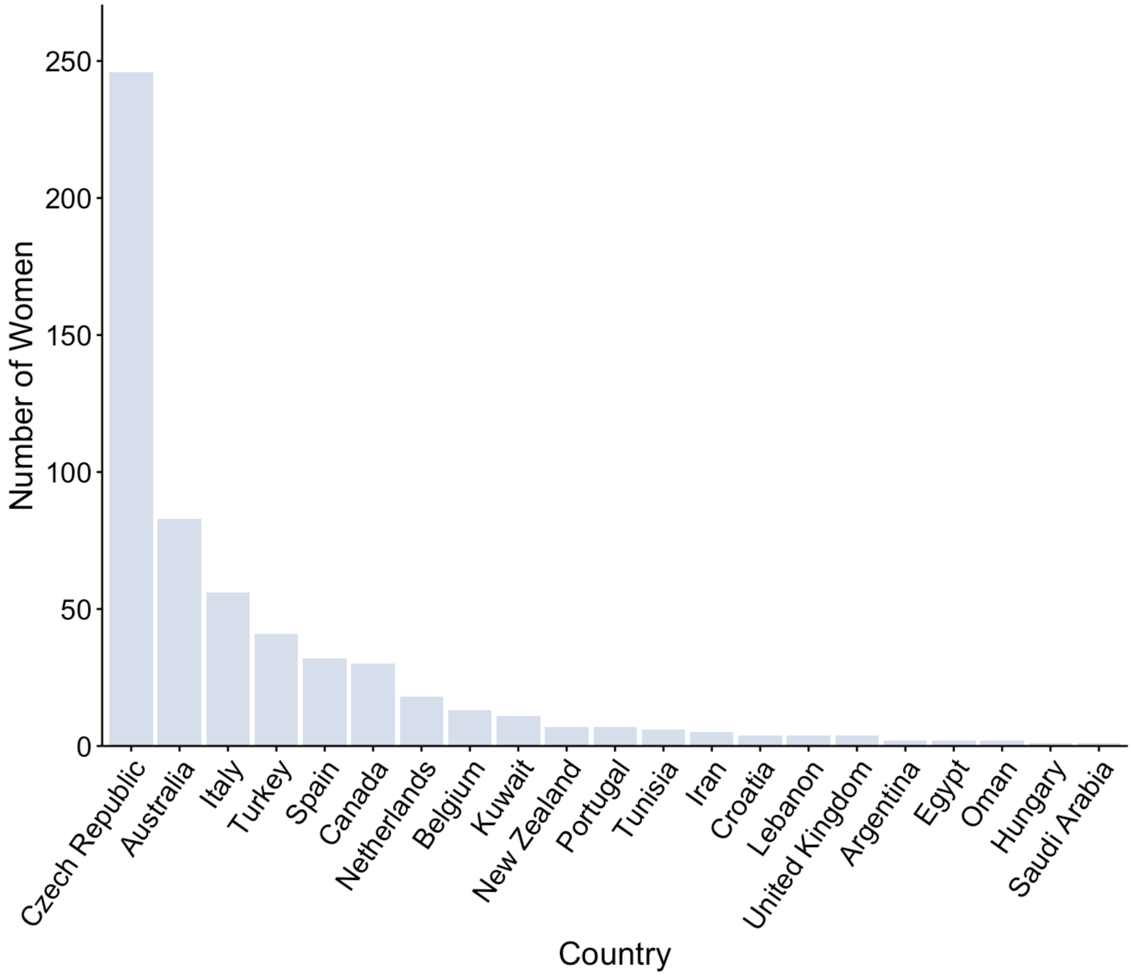

© 2025 Shipley J et al. *JAMA Network Open*

eFigure 3. Preconception EDSS Scores of Women in the Pregnant Cohort

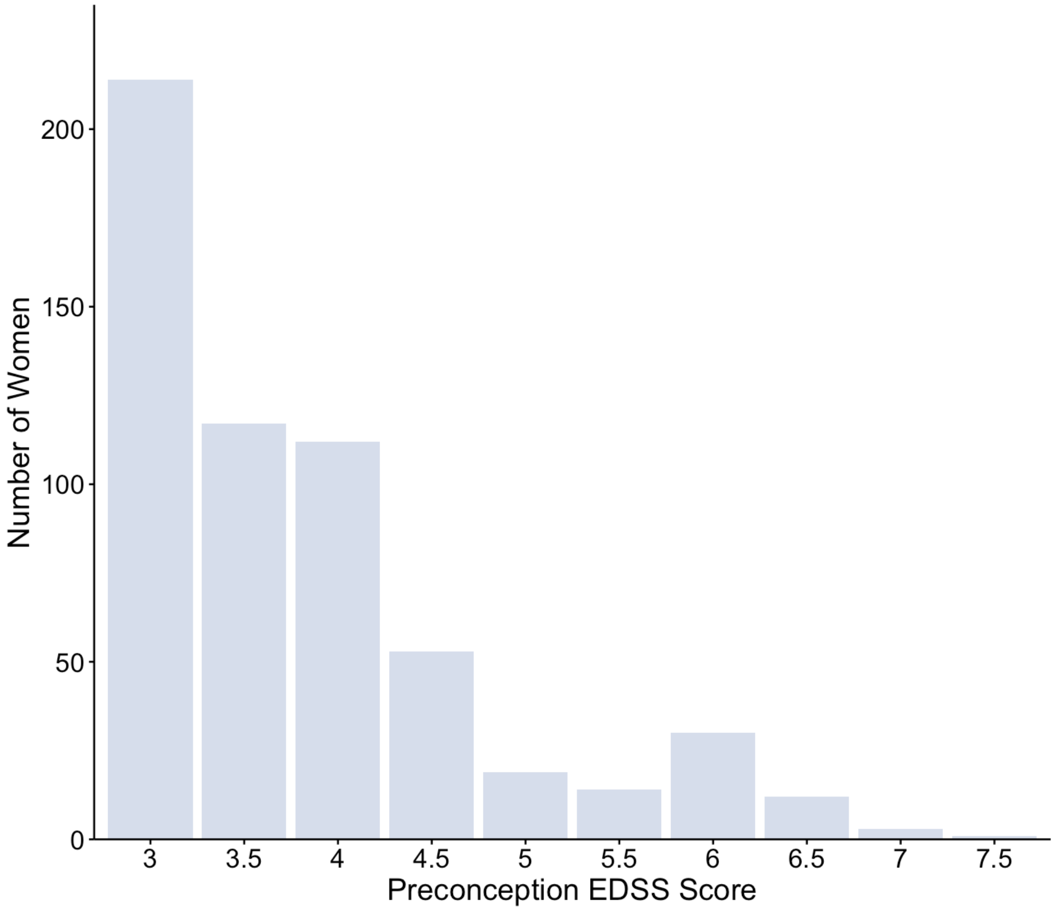

© 2025 Shipley J et al. *JAMA Network Open*

eTable 2. Washout Periods of Preconception Disease-Modifying Therapies

DMT Type	n (n = 575)	Months Before Pregnancy, median (IQR)
**DMTs used in the preconception year**	469	-0.99 (-1.77-0.39)
**Interferon**	141	-1.12 (-1.94-0.00)
**Natalizumab**	104	-1.07 (-2.78--0.07)
**Glatiramer acetate**	76	-0.99 (-1.54-0.00)
**Fingolimod**	46	-0.59 (-1.32-2.19)
**Anti-CD20 therapy**	40[a]	0.84 (-0.90-3.43)
**Dimethyl fumarate**	31	-1.18 (-1.84--0.76)
**Alemtuzumab**	9	7.00 (1.84-8.94)
**Teriflunomide**	8	-0.87 (-1.27-2.01)
**Cladribine**	4	8.80 (6.82-9.17)

Abbreviations: DMT, disease-modifying therapy; IQR, interquartile range.
For the most common DMTs used in the 12 months prior to conception. For individuals who used more than one DMT type in the preconception year, only the DMT commenced closest to conception was included. Positive values indicate withdrawal of the DMT prior to pregnancy, and negative values indicate continuation after conception. In some cases, a DMT may have been withdrawn for pregnancy, but the temporary discontinuation and reinitiation dates may not have been entered in the registry, which may influence the accuracy of the data.
[a] Ocrelizumab (n = 33), rituximab (n = 7)

© 2025 Shipley J et al. *JAMA Network Open*

eTable 3. Timing of Reinitiation of Disease-Modifying Therapies After Pregnancy

DMT Type	n (n = 575)	Months to Reinitiation, median (IQR)
**DMT used in the postpartum year**	438	1.46 (0.00-3.90)
**Interferon**	131	2.43 (0.34-5.17)
**Natalizumab**	103	0.56 (0.00-1.69)
**Glatiramer acetate**	62	1.84 (0.01-5.89)
**S1P receptor modulator**	50[a]	1.61 (0.11-3.49)
**Anti-CD20 therapy**	36[b]	1.46 (0.07-3.60)
**Dimethyl fumarate**	31	2.17 (0.77-3.12)
**Alemtuzumab**	9	1.54 (0.85-2.33)
**Teriflunomide**	8	1.58 (0.39-2.67)
**Cladribine**	4	7.79 (5.69-8.63)
**Mitoxantrone**	2	4.01 (2.30-5.72)
**Cyclophosphamide**	1	7.88 (7.88-7.88)

Abbreviations: DMT, disease-modifying therapy; IQR, interquartile range; S1P, sphingosine-1-phosphate. For the most common DMTs reinitiated within 12 months postpartum. For individuals who used more than one DMT type in the postpartum year, only the first DMT initiated after pregnancy was included. The reinitiation time was set to 0 if a DMT was continued during pregnancy without an interval off treatment, or if it was recommenced prior to the end date of pregnancy.

[a] Fingolimod (n = 48), ozanimod (n = 1), siponimod (n = 1)

[b] Ocrelizumab (n = 29), rituximab (n = 6), ofatumumab (n = 1)

© 2025 Shipley J et al. *JAMA Network Open*

eFigure 4. Annualised Relapse Rates and Rate Ratios for First-Trimester Pregnancy Losses

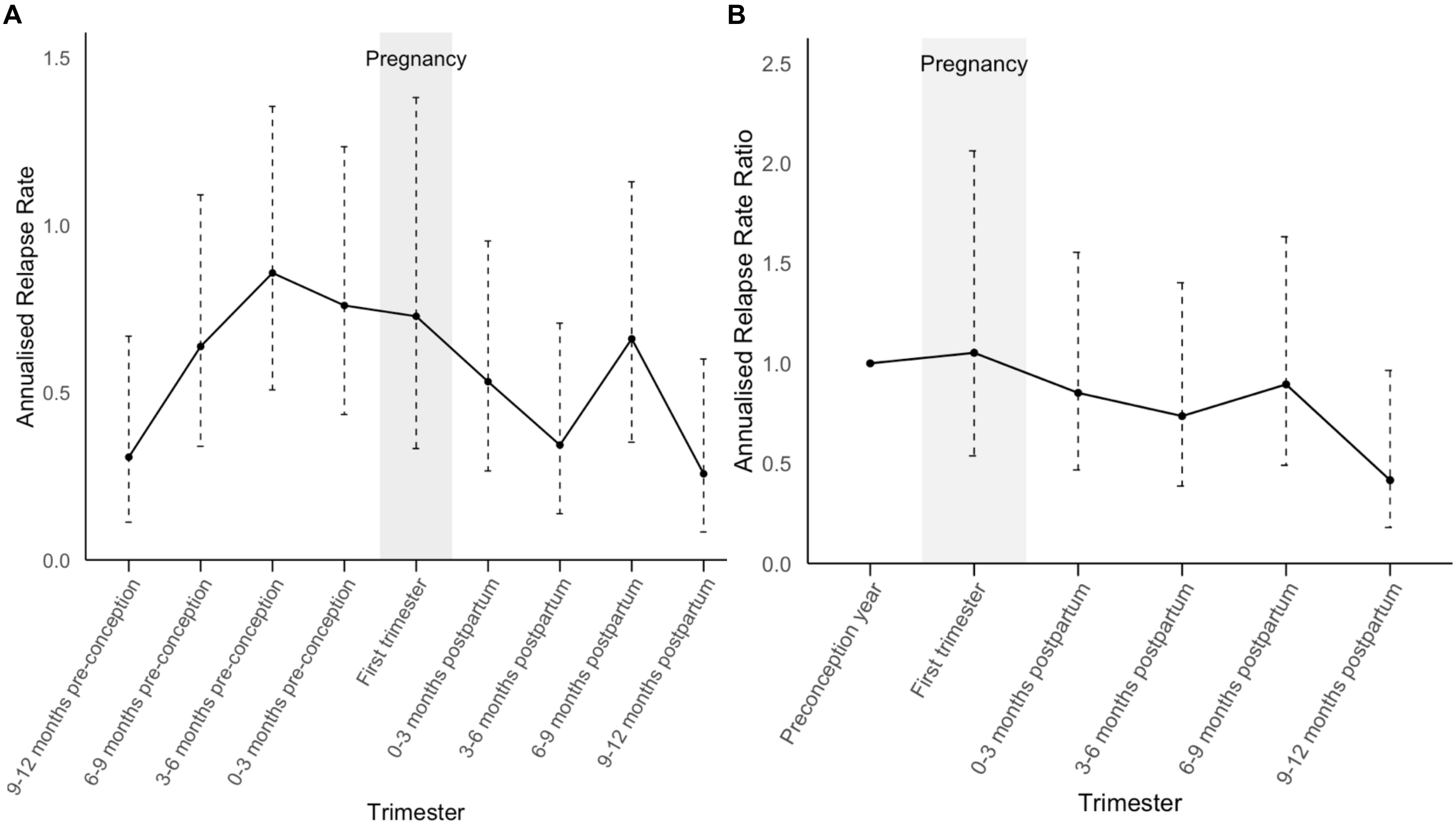

n = 85 first-trimester pregnancy losses (miscarriage or termination)
A: Absolute peri-pregnancy annualised relapse rates; B: Rate ratios for relapses relative to the preconception year calculated using a mixed-effects Poisson regression model

© 2025 Shipley J et al. *JAMA Network Open*

eFigure 5. Annualised Relapse Rates and Rate Ratios for First-Trimester and Second-Trimester Pregnancy Losses

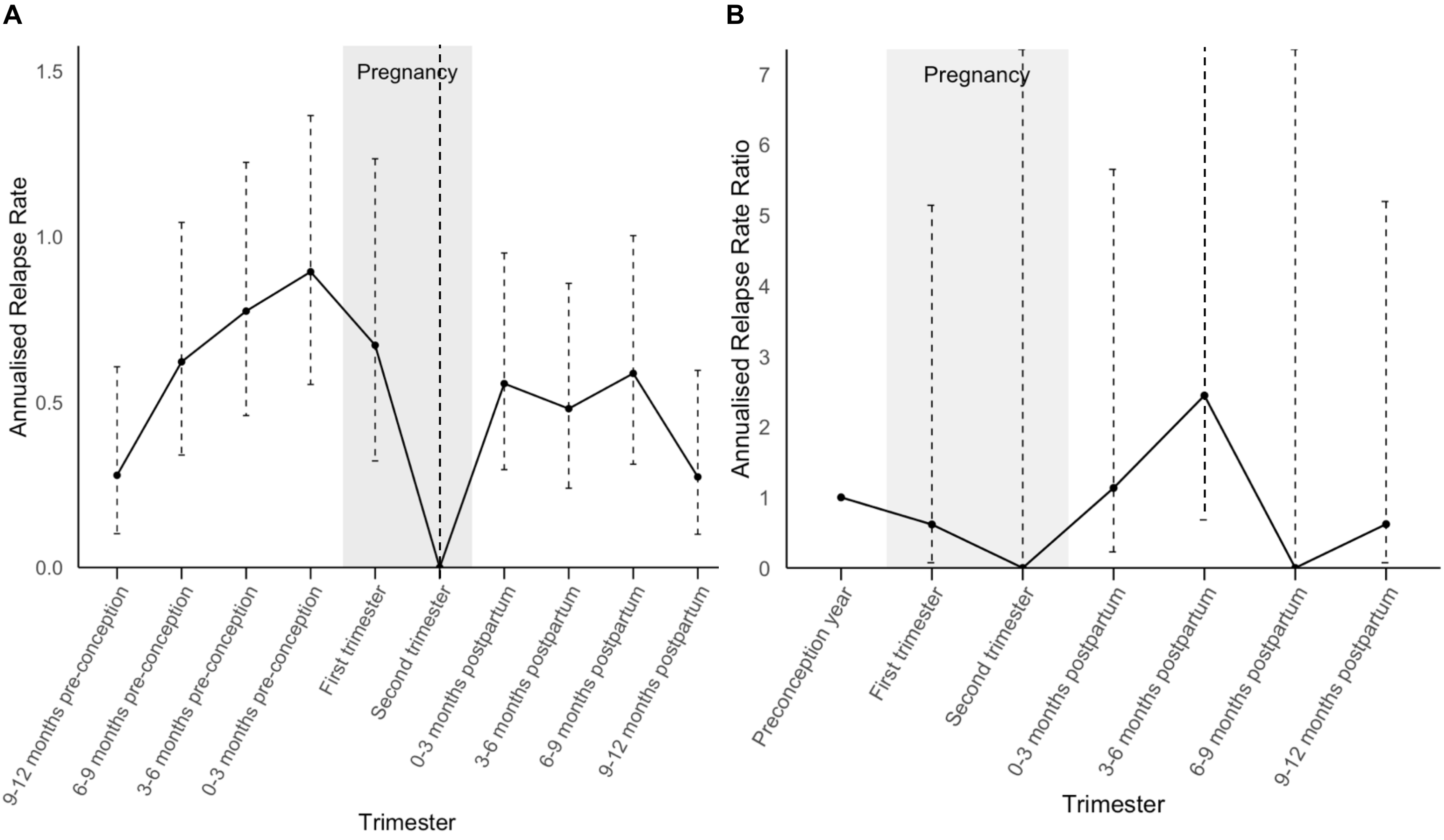

n = 85 first-trimester pregnancy losses (miscarriage or termination) and n = 11 second-trimester pregnancy losses. There were no reported events during the second trimester.

A: Absolute peri-pregnancy annualised relapse rates; B: Rate ratios for relapses relative to the preconception year calculated using a mixed-effects Poisson regression model

eTable 4. Annualised Relapse Rate Ratios for Live Births by Disease-Modifying Therapy and Treatment Epoch

Category	n	Time Period	Annualised Relapse Rate Ratio (95% CI)[a]	P value
**All live births**	469	Preconception year	1.00	-
		First trimester	0.25 (0.15-0.43)	<0.001
		Second trimester	0.41 (0.28-0.60)	<0.001
		Third trimester	0.36 (0.23-0.56)	<0.001
		0-3 months postpartum	1.36 (1.06-1.75)	0.01
		3-6 months postpartum	0.79 (0.58-1.08)	0.14
		6-9 months postpartum	1.01 (0.76-1.34)	0.94
		9-12 months postpartum	0.88 (0.65-1.19)	0.39
**Live births, excluding rebound disease activity[b]**	346	Preconception year	1.00	-
		First trimester	0.20 (0.10-0.40)	<0.001
		Second trimester	0.21 (0.12-0.39)	<0.001
		Third trimester	0.15 (0.07-0.32)	<0.001
		0-3 months postpartum	1.39 (1.05-1.85)	0.02
		3-6 months postpartum	0.86 (0.61-1.22)	0.40
		6-9 months postpartum	0.95 (0.68-1.33)	0.76
		9-12 months postpartum	1.01 (0.73-1.41)	0.94
**Most common preconception DMTs**				
**Interferon**	117	Preconception year	1.00	-
		First trimester	0.22 (0.08-0.61)	0.004
		Second trimester	0.29 (0.12-0.66)	0.003
		Third trimester	0.21 (0.08-0.59)	0.003
		0-3 months postpartum	1.57 (1.02-2.40)	0.04
		3-6 months postpartum	1.05 (0.64-1.73)	0.84
		6-9 months postpartum	1.20 (0.74-1.93)	0.46
		9-12 months postpartum	1.33 (0.84-2.11)	0.23
**Natalizumab**	84	Preconception year	1.00	-
		First trimester	0.10 (0.01-0.71)	0.02
		Second trimester	0.75 (0.37-1.55)	0.44
		Third trimester	0.59 (0.25-1.37)	0.22
		0-3 months postpartum	0.83 (0.40-1.69)	0.60
		3-6 months postpartum	0.55 (0.24-1.30)	0.18
		6-9 months postpartum	0.76 (0.36-1.61)	0.48
		9-12 months postpartum	0.49 (0.19-1.24)	0.13
**Glatiramer acetate**	63	Preconception year	1.00	-
		First trimester	0.28 (0.07-1.17)	0.08
		Second trimester	0.36 (0.11-1.18)	0.09
		Third trimester	0.00 (0.00-Inf)	0.92
		0-3 months postpartum	1.41 (0.71-2.83	0.33
		3-6 months postpartum	0.64 (0.25-1.66)	0.36
		6-9 months postpartum	0.90 (0.39-2.05)	0.80
		9-12 months postpartum	1.03 (0.47-2.25)	0.95
**Fingolimod**	31	Preconception year	1.00	-
		First trimester	0.94 (0.32-2.79)	0.92
		Second trimester	1.01 (0.38-2.73)	0.98
		Third trimester	2.23 (1.03-4.83)	0.04
		0-3 months postpartum	1.74 (0.76-4.00)	0.19
		3-6 months postpartum	1.10 (0.41-2.97)	0.85
		6-9 months postpartum	1.56 (0.65-3.74)	0.32
		9-12 months postpartum	0.23 (0.03-1.72)	0.15
**Anti-CD20 therapy**	31[c]	Preconception year	1.00	-
		First trimester	0.00 (0.00-Inf)	1.00
		Second trimester	0.00 (0.00-Inf)	1.00
		Third trimester	0.00 (0.00-Inf)	1.00
		0-3 months postpartum	0.75 (0.15-3.71)	0.72
		3-6 months postpartum	0.42 (0.05-3.73)	0.44

© 2025 Shipley J et al. *JAMA Network Open*

		6-9 months postpartum	0.00 (0.00-Inf)	1.00
		9-12 months postpartum	0.95 (0.19-4.71)	0.95
**Dimethyl fumarate**	27	Preconception year	1.00	-
		First trimester	0.00 (0.00-Inf)	0.97
		Second trimester	0.07 (0.04-2.77)	0.32
		Third trimester	0.00 (0.00-Inf)	0.98
		0-3 months postpartum	2.30 (0.84-6.34)	0.11
		3-6 months postpartum	0.40 (0.05-3.08)	0.38
		6-9 months postpartum	0.79 (0.17-3.60)	0.76
		9-12 months postpartum	0.79 (0.17-3.60)	0.76
**None**	89	Preconception year	1.00	-
		First trimester	0.25 (0.08-0.81)	0.02
		Second trimester	0.07 (0.01-0.52)	0.009
		Third trimester	0.16 (0.04-0.65)	0.01
		0-3 months postpartum	1.24 (0.70-2.20)	0.46
		3-6 months postpartum	0.88 (0.45-1.71)	0.71
		6-9 months postpartum	0.97 (0.51-1.84)	0.93
		9-12 months postpartum	0.74 (0.36-1.53)	0.42
**Natalizumab strategy**				
**Natalizumab discontinued preconception**	23	Preconception year	1.00	-
		First trimester	0.00 (0.00-Inf)	1.00
		Second trimester	1.24 (0.44-3.52)	0.68
		Third trimester	0.96 (0.27-3.43)	0.95
		0-3 months postpartum	1.07 (0.34-3.32)	0.91
		3-6 months postpartum	0.55 (0.12-2.50)	0.44
		6-9 months postpartum	1.39 (0.49-3.95)	0.53
	42	9-12 months postpartum	1.15 (0.37-3.59)	0.81
**Natalizumab discontinued during the first or second trimester**		Preconception year	1.00	-
		First trimester	0.23 (0.03-1.70)	0.15
		Second trimester	0.78 (0.27-2.29)	0.65
		Third trimester	0.43 (0.10-1.85)	0.26
		0-3 months postpartum	0.88 (0.30-2.57)	0.81
		3-6 months postpartum	0.44 (0.10-1.88)	0.27
		6-9 months postpartum	0.66 (0.19-2.22)	0.50
		9-12 months postpartum	0.22 (0.03-1.67)	0.14
**Natalizumab continued into the third trimester**	19	Preconception year	1.00	-
		First trimester	0.00 (0.00-Inf)	1.00
		Second trimester	0.00 (0.00-Inf)	1.00
		Third trimester	0.41 (0.05-3.25)	0.40
		0-3 months postpartum	0.39 (0.05-3.08)	0.37
		3-6 months postpartum	0.25 (0.17-3.59)	0.75
		6-9 months postpartum	0.00 (0.00-Inf)	1.00
		9-12 months postpartum	0.00 (0.00-Inf)	1.00
**Treatment epoch[d]**				
**Modern epoch**	343	Preconception year	1.00	-
		First trimester	0.22 (0.11-0.44)	<0.001
		Second trimester	0.49 (0.31-0.77)	0.002
		Third trimester	0.49 (0.31-0.79)	0.004
		0-3 months postpartum	1.54 (1.14-2.07)	0.005
		3-6 months postpartum	0.82 (0.55-1.20)	0.31
		6-9 months postpartum	0.89 (0.61-1.30)	0.55
		9-12 months postpartum	0.77 (0.52-1.16)	0.21
**Middle epoch**	98	Preconception year	1.00	-
		First trimester	0.24 (0.09-0.65)	0.005
		Second trimester	0.30 (0.13-0.70)	0.005
		Third trimester	0.18 (0.06-0.56)	0.003
		0-3 months postpartum	1.14 (0.70-1.86)	0.61
		3-6 months postpartum	0.87 (0.51-1.51)	0.63
		6-9 months postpartum	1.11 (0.67-1.84)	0.68
		9-12 months postpartum	1.02 (0.60-1.71)	0.95

© 2025 Shipley J et al. *JAMA Network Open*

**Early epoch**	28	Preconception year	1.00	-
		First trimester	0.55 (0.16-1.85)	0.33
		Second trimester	0.16 (0.02-1.17)	0.07
		Third trimester	0.00 (0.00-Inf)	0.97
		0-3 months postpartum	0.84 (0.32-2.24)	0.73
		3-6 months postpartum	0.34 (0.08-1.44)	0.14
		6-9 months postpartum	1.35 (0.60-3.05)	0.47
		9-12 months postpartum	1.01 (0.41-2.51)	0.98

Abbreviations: CI, confidence interval; DMT, disease-modifying therapy.
Infinite CIs were generated for certain time intervals due to no recorded events, preventing the model from estimating finite coefficients.

[a] Relative to the preconception year

[b] Excluding fingolimod use and natalizumab discontinuation prior to the third trimester of pregnancy

[c] Ocrelizumab (n = 25), rituximab (n = 6)

[d] The early epoch was defined as the period before 2005, the middle epoch as 2005 to 2010, and the modern epoch as the period after 2010

© 2025 Shipley J et al. *JAMA Network Open*

eFigure 6. Annualised Relapse Rates for Live Births by Disease-Modifying Therapy and Treatment Epoch

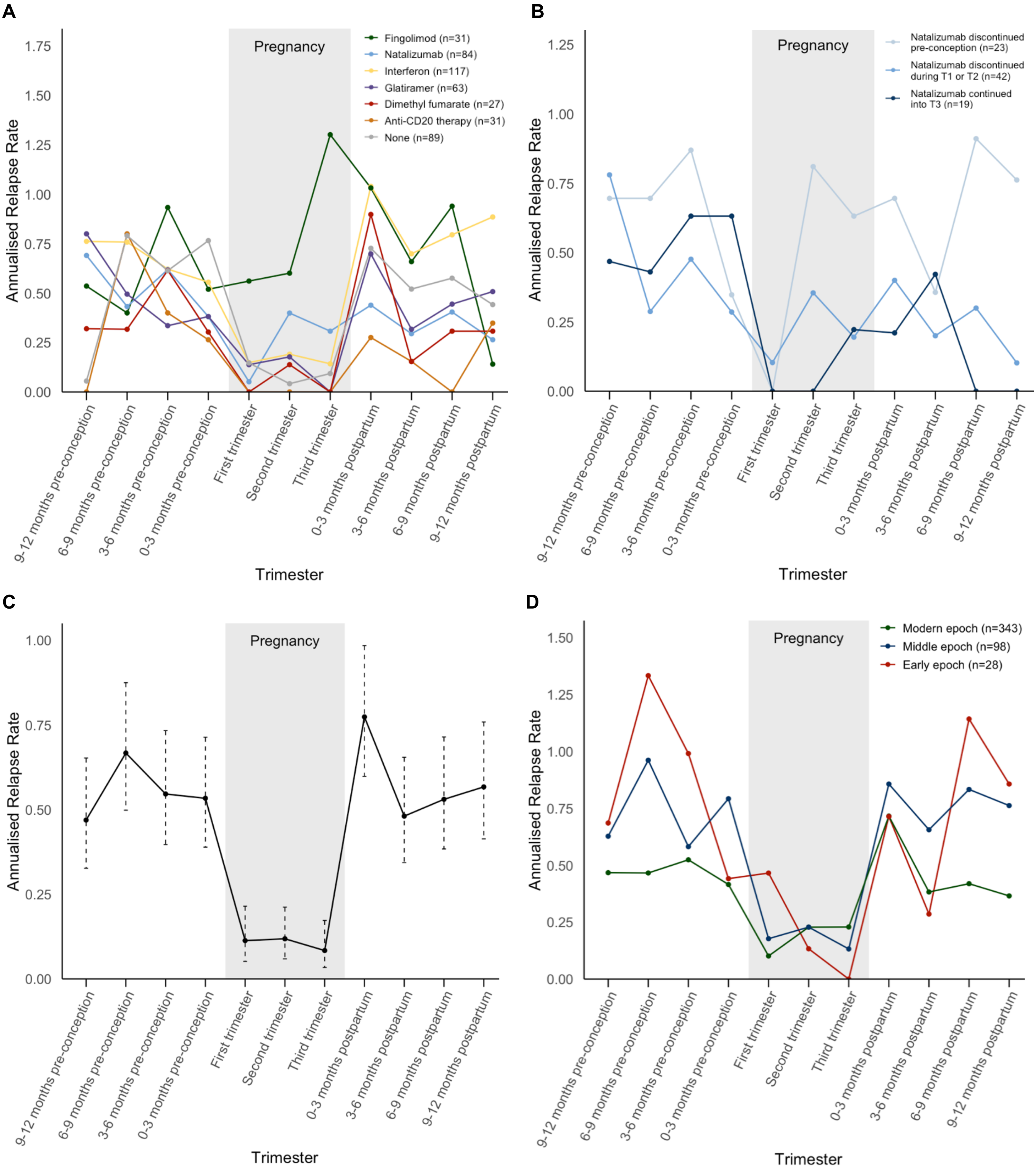

Abbreviations: T1, first trimester of pregnancy; T2, second trimester of pregnancy; T3, third trimester of pregnancy.
A: Peri-pregnancy annualised relapse rates (ARRs) by most common preconception DMTs. Anti-CD20 therapy included ocrelizumab (n = 25) or rituximab (n = 6); B: Peri-pregnancy ARRs by natalizumab prescribing strategy; C: Peri-pregnancy ARRs for live births, excluding the rebound effect associated with fingolimod use or natalizumab

© 2025 Shipley J et al. *JAMA Network Open*

discontinuation prior to the third trimester (n = 346); D: Peri-pregnancy ARRs by treatment epoch. The early epoch was defined as the period before 2005, the middle epoch as 2005 to 2010, and the modern epoch as the period after 2010

© 2025 Shipley J et al. *JAMA Network Open*

eTable 5. Covariate Balance Between Pregnant and Non-Pregnant Cohorts Before and After 2:1 Propensity Score Matching

Covariate	Type	Unadj. Mean (SD)		Unadj. SMD	Balance Status Pre-Matching[a]	Adj. Mean (SD)		Adj. SMD	Balance Status Post-Matching
		Pregnant (n = 528)	Non-pregnant (n = 5419)			Pregnant (n = 528)	Non-pregnant (n = 1056)		
**Distance**				0.729	Not balanced			0.004	*Balanced*
**MS phenotype[b]**	Binary	-	-	0.041	*Balanced*	-	-	-0.002	*Balanced*
**Age at baseline**	Cont	32.38 (4.91)	36.58 (6.24)	-0.748	Not balanced	32.38 (4.91)	32.38 (6.03)	0.002	*Balanced*
**Disease duration at baseline**	Cont	8.86 (5.48)	8.72 (6.25)	0.024	*Balanced*	8.86 (5.48)	9.00 (6.14)	-0.039	*Balanced*
**Baseline EDSS**	Cont	3.81 (0.96)	4.19 (1.23)	-0.345	Not balanced	3.81 (0.96)	3.83 (0.99)	-0.021	*Balanced*
**ARR in the pre-baseline year**	Cont	0.56 (0.81)	0.76 (1.10)	-0.216	Not balanced	0.56 (0.81)	0.56 (0.85)	-0.008	*Balanced*
**Use of high-efficacy DMT in the pre-baseline year[c]**	Binary	-	-	0.119	Not balanced	-	-	0.015	*Balanced*

Abbreviations: Adj, adjusted; ARR, annualised relapse rate; cont, continuous; DMT, disease-modifying therapy; EDSS, Expanded Disability Status Scale; SD, standard deviation; SMD, standardized mean difference; unadj, unadjusted.

[a] A variable was considered balanced if the absolute SMD was less than 0.1

[b] Relapse-onset or progressive-onset

[c] High-efficacy DMT was defined as alemtuzumab, anti-CD20 monoclonal antibodies, natalizumab, cladribine, sphingosine-1-phosphate receptor inhibitors, cyclophosphamide, and autologous haematopoietic stem cell transplant

© 2025 Shipley J et al. *JAMA Network Open*

eFigure 7. Covariate Balance Between Pregnant and Non-Pregnant Cohorts Before and After 2:1 Propensity Score Matching

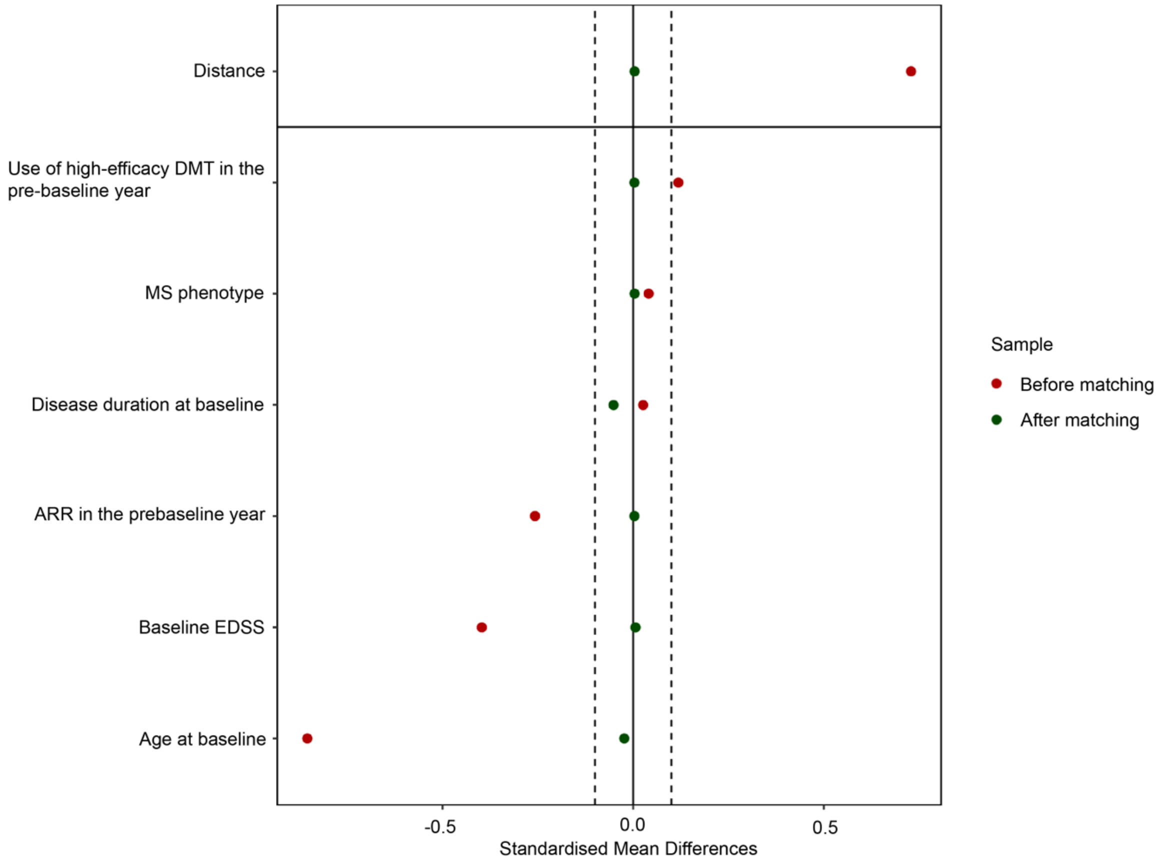

Abbreviations: ARR, annualised relapse rate; DMT, disease-modifying therapy; EDSS, Expanded Disability Status Scale.
Dashed vertical lines represent the commonly accepted threshold values of ±0.1.

© 2025 Shipley J et al. *JAMA Network Open*

eTable 6. Survival Analysis of Time to 6-Month Confirmed Disability Worsening for All Pregnancies

Covariate	Unadj. HR (95% CI 0.67-1.05)	Unadj. P value	Adj. HR (95% CI 0.67-1.05)	Adj. P value
Pregnancy history	1.15 (0.96-1.38)	0.13	1.15 (0.96-1.38)	0.13
High-efficacy DMT exposure (time-varying covariate)	-	-	1.00 (0.90-1.12)	0.98

Abbreviations: Adj, adjusted; CI, confidence interval; DMT, disease-modifying therapy; HR, hazard ratio; unadj, unadjusted.
Comparing women with MS with pregnancies of any duration and meeting other inclusion criteria for the survival analysis (n = 528) and a non-pregnant cohort (n = 1056) matched with 2:1 propensity score matching.

© 2025 Shipley J et al. *JAMA Network Open*

eTable 7. Assessment of Proportional Hazards Assumption Using Schoenfeld Residuals

Covariate	Chi-square	Degrees of Freedom	P value
Pregnancy history	0.002	1	0.96
High-efficacy DMT exposure (time-varying covariate)	0.054	1	0.82
Global	0.055	2	0.97

Abbreviations: DMT, disease-modifying therapy.
Results of the proportional hazards assumption test using Schoenfeld residuals for the Cox regression model assessing the association between pregnancy history and time to 6-month confirmed disability worsening. A non-significant P value indicates that the proportional hazards assumption is not violated for the corresponding covariate or the overall model.

© 2025 Shipley J et al. *JAMA Network Open*

eFigure 8. Assessment of Proportional Hazards Assumption Using Scaled Schoenfeld Residual Plots

A

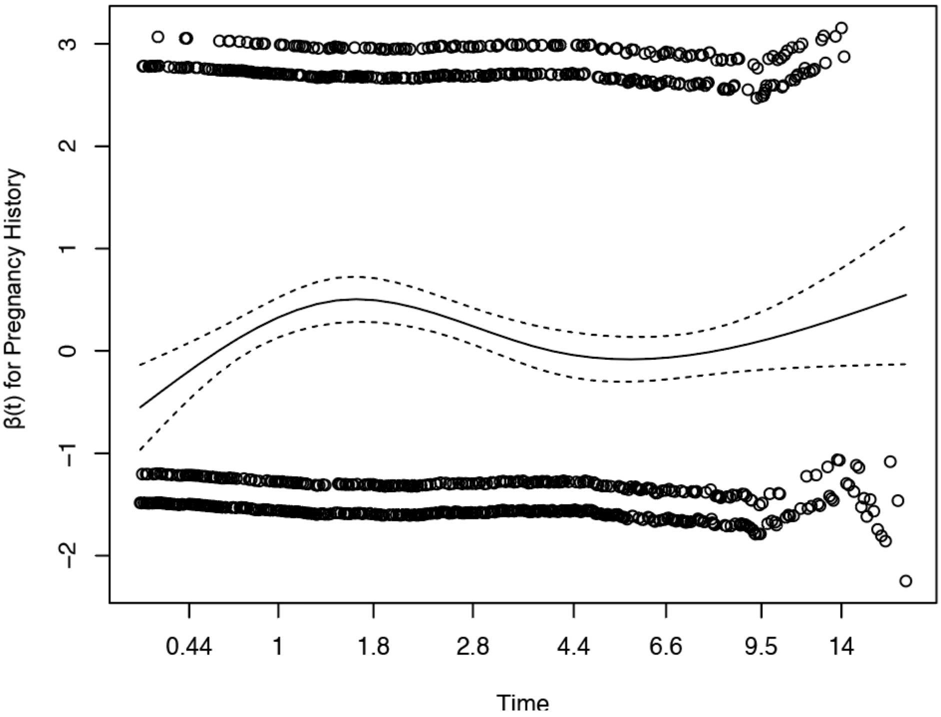

B

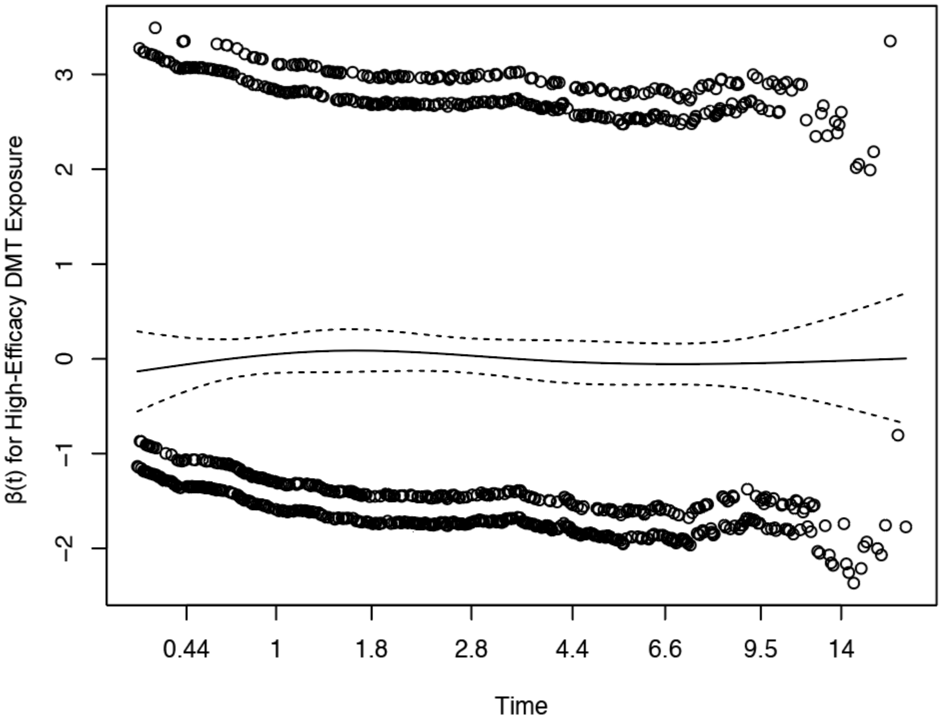

Abbreviations: DMT, disease-modifying therapy.
A: Scaled Schoenfeld residual plot for pregnancy history; B: Scaled Schoenfeld residual plot for high-efficacy DMT exposure as a time-varying covariate

© 2025 Shipley J et al. *JAMA Network Open*

eTable 8. Predictors of Time to 6-Month Confirmed Disability Worsening for Pregnant Women with Live Births

Variable	n (n = 439)	Unadj. HR (95% CI)	Unadj. P value	Adj. HR (95% CI)	Adj. P value
**Age at conception**		1.01 (0.98-1.04)	0.46	1.00 (0.96-1.04)	0.89
**Disease duration at conception**		1.00 (0.98-1.03)	0.88	0.98 (0.94-1.02)	0.32
**Preconception EDSS >4**	94	1.68 (1.23-2.30)	0.001	0.94 (0.61-1.45)	0.78
**ARR in the preconception year**		1.18 (1.01-1.39)	0.04	1.20 (0.99-1.47)	0.06
**ARR during pregnancy**		1.40 (1.15-1.71)	0.001	1.37 (1.13-1.65)	0.001
**DMT continuation into pregnancy**	219	0.70 (0.53-0.93)	0.01	0.74 (0.53-1.05)	0.09
**Monoclonal antibody therapy in the preconception year**	116	1.15 (0.83-1.61)	0.40	NA	NA
**First postpartum EDSS >4**	114	2.49 (1.87-3.32)	<0.001	2.69 (1.80-4.03)	<0.001
**ARR in the first 6 months postpartum**		1.17 (1.04-1.31)	0.008	1.10 (0.96-1.26)	0.16
**Time to DMT reinitiation**	344	1.00 (1.00-1.00)	0.86	1.00 (1.00-1.00)	0.93
**Monoclonal antibody therapy in the postpartum year**	108	1.10 (0.78-1.55)	0.60	NA	NA
**Breastfeeding duration**	182	0.93 (0.88-0.98)	0.01	0.93 (0.85-1.01)	0.08

Abbreviations: Adj, adjusted; ARR, annualised relapse rate; CI, confidence interval; DMT, disease-modifying therapy; EDSS, Expanded Disability Status Scale; HR, hazard ratio; unadj, unadjusted.

eTable 9. Predictors of Relapse During Pregnancy for Live Births in the Modern Epoch

Variable	n (n = 343)	Unadj. OR (95% CI)	Unadj. P value	Adj. OR (95% CI)	Adj. P value
**Age at conception**		0.93 (0.86-1.00)	0.05	0.87 (0.79-0.96)	0.005
**Disease duration at conception**		1.05 (0.99-1.11)	0.14	1.13 (1.04-1.23)	0.004
**Preconception EDSS >4**	73	0.98 (0.43-2.25)	0.97	0.87 (0.33-2.09)	0.76
**ARR in the preconception year**		1.63 (1.08-2.46)	0.02	1.60 (1.00-2.54)	0.05
**DMT used within 1 month of conception[a]**					
**Natalizumab**	75	15.34 (1.93-1983.83)	0.005	13.88 (1.69-1807.66)	0.009
**Interferon**	62	8.17 (0.92-1077.4)	0.06	7.65 (0.82-1017.75)	0.08
**Anti-CD20 therapy[b]**	27	1.29 (0.01-241.84)	0.90	0.74 (0.00-141.93)	0.88
**Dimethyl fumarate**	24	4.53 (0.23-673.01)	0.32	8.87 (0.44-1341.29)	0.15
**Fingolimod**	16	33.96 (3.43-4587.97)	0.001	51.13 (4.73-7070.63)	<0.001
**None**	94	10.76 (1.35-1392.22)	0.02	4.53 (0.50-603.29)	0.22
**Monoclonal antibody therapy in the preconception year**	122	1.06 (0.53-2.14)	0.86	NA	NA
**Natalizumab exposure relative to conception**					
**Natalizumab discontinued preconception**	20	6.83 (1.20-39.05)	0.03	NA	NA
**Natalizumab discontinued during T1 or T2**	41	4.22 (0.82-21.67)	0.08	NA	NA
**Natalizumab use during T3**	19	1.14 (0.10-13.38)	0.92	NA	NA
**DMT washout period**	88	1.00 (0.99-1.01)	0.31	NA	NA
**DMT continuation into pregnancy**	190	0.55 (0.28-1.08)	0.08	0.29 (0.11-0.78)	0.01

Abbreviations: Adj, adjusted; ARR, annualised relapse rate; CI, confidence interval; DMT, disease-modifying therapy; EDSS, Expanded Disability Status Scale; OR, odds ratio; unadj, unadjusted.
Glatiramer acetate was designated as the reference DMT for DMT variables.
[a] DMT administration dates within 1 month of conception, or estimated infusion date within 6 months of conception for anti-CD20 therapies
[b] Ocrelizumab (n = 21), rituximab (n = 6). Firth's correction was used in both the univariable and multivariable models to generate confidence intervals for anti-CD20 therapies due to complete separation caused by the absence of relapses during pregnancy in this group

© 2025 Shipley J et al. *JAMA Network Open*

eTable 10. Predictors of Relapse Within 3 Months Postpartum for Live Births in the Modern Epoch

Variable	n (n = 343)	Unadj. OR (95% CI)	Unadj. P value	Adj. OR (95% CI)	Adj. P value
**Age at conception**		0.97 (0.91-1.03)	0.33	0.98 (0.91-1.06)	0.67
**Disease duration at conception**		0.97 (0.92-1.03)	0.33	0.99 (0.92-1.05)	0.68
**Preconception EDSS >4**	73	0.84 (0.41-1.72)	0.64	1.02 (0.46-2.23)	0.96
**ARR in the preconception year**		1.29 (0.89-1.87)	0.18	1.25 (0.83-1.87)	0.29
**ARR during pregnancy**		1.28 (0.87-1.87)	0.21	1.18 (0.76-1.84)	0.47
**DMT continuation into pregnancy**	190	0.91 (0.52-1.6)	0.74	0.83 (0.44-1.58)	0.58
**Natalizumab exposure relative to conception**					
**Natalizumab discontinued preconception**	20	0.67 (0.16-2.79)	0.58	NA	NA
**Natalizumab discontinued during T1 or T2**	41	0.3 (0.07-1.19)	0.09	NA	NA
**Natalizumab use during T3**	19	0.21 (0.03-1.79)	0.15	NA	NA
**Pregnancy duration**		1.00 (0.98-1.02)	0.85	NA	NA
**Breastfeeding**	154	1.00 (0.57-1.76)	0.99	0.83 (0.44-1.59)	0.58
**DMT reinitiation within 1 month**	103	0.45 (0.23-0.89)	0.02	0.43 (0.20-0.91)	0.03
**Monoclonal antibody therapy reinitiated within 1 month postpartum**	54	0.45 (0.17-1.2)	0.11	NA	NA
**Timing of natalizumab reinitiation**					
**Early natalizumab reinitiation (≤14 days postpartum)**	27	0.42 (0.07-2.35)	0.32	NA	NA
**Late natalizumab reinitiation (>14 days postpartum)**	49	1.17 (0.35-3.88)	0.80	NA	NA

Abbreviations: Adj, adjusted; ARR, annualised relapse rate; CI, confidence interval; DMT, disease-modifying therapy; EDSS, Expanded Disability Status Scale; OR, odds ratio; T1, first trimester of pregnancy; T2, second trimester of pregnancy; T3, third trimester of pregnancy; unadj, unadjusted.
Glatiramer acetate was designated as the reference DMT for DMT variables.

© 2025 Shipley J et al. *JAMA Network Open*

eTable 11. Survival Analysis of Time to 6-Month Confirmed Disability Worsening for Live Births Only

Covariate	Unadj. HR (95% CI 0.67-1.05)	Unadj. P value	Adj. HR (95% CI 0.67-1.05)	Adj. P value
Pregnancy history	0.88 (0.70-1.10)	0.25	1.10 (0.91-1.33)	0.33
High-efficacy DMT exposure (time-varying covariate)	-	-	0.93 (0.82-1.05)	0.21

Abbreviations: Adj, adjusted; CI, confidence interval; DMT, disease-modifying therapy; HR, hazard ratio; unadj, unadjusted.
Comparing women with MS with live births and meeting other inclusion criteria for the survival analysis (n = 439) and a non-pregnant cohort (n = 878) matched with 2:1 propensity score matching.
