# Supplementary material for: Pregnancy-Related Disease Outcomes in Women With Moderate to Severe Multiple Sclerosis Disability
Source: JAMA Netw Open. 2025 Sep 15;8(9):e2531581. doi: 10.1001/jamanetworkopen.2025.31581 (PMC12439057; doi:10.1001/jamanetworkopen.2025.31581)
Supplement: Supplement 2. — Nonauthor Collaborators [file jamanetwopen-e2531581-s002.pdf]

Supplemental Online Content: Nonauthor Collaborators
*First name, last name, and suffix (if applicable) are required and will appear in PubMed.

***Group Name(s): MSBase Investigator Group**

***First Name and Middle Initial(s)**	***Last Name**	***Suffix (eg, Jr, III)**	Academic Degrees	Institution	Location (city, state/province, country)	Role or Contribution, eg, chair, principal investigator	Group (if more than 1 Group listed in the byline) and/or Subgroup (eg, Steering Committee)
Marta	Varchova		MD	Department of Neurology, KZ a.s., Teplice Hospital	Teplice, Czech Republic	Data contributor	
Pierre	Duquette		MD	Centre hospitalier de l'Université de Montréal (CHUM) and Université de Montréal	Montreal, Canada	Data contributor	
Jana	Libertinova		MD, PhD	Department of Neurology, Second Faculty of Medicine, Charles University and Motol University Hospital	Prague, Czech Republic	Data contributor	
Guillermo	Izquierdo		MD	Department of Neurology, Hospital Universitario Virgen Macarena	Seville, Spain	Data contributor	
Sara	Eichau		MD	Department of Neurology, Hospital Universitario Virgen Macarena	Seville, Spain	Data contributor	
Francesco	Patti		MD	Department of Medical and Surgical Sciences and Advanced Technologies, G.F. Ingrassia	Catania, Italy	Data contributor	
Cavit	Boz		MD	Department of Neurology, Medical Faculty, Karadeniz Technical University	Trabzon, Turkey	Data contributor	
Michael	Barnett		MBBS, PhD	Brain and Mind Centre	Sydney, Australia	Data contributor	
Radek	Ampapa		MD, PhD	Nemocnice Jihlava	Jihlava, Czech Republic	Data contributor	
Alena	Martinkova		MD	Department of Neurology, Hospital Pardubice	Pardubice, Czech Republic	Data contributor	
Cristina	Ramo-Tello		MD, PhD	Department of Neuroscience, Hospital Germans Trias i Pujol	Badalona, Spain	Data contributor	
Pierre	Grammond		MD	CISSS de Chaudière-Appalaches	Lévis, Canada	Data contributor	
Vincent	van Pesch		MD	Department of Neurology, Cliniques Universitaires Saint-Luc	Brussels, Belgium	Data contributor	
MSBase Foundation Centre Custodian				University Hospital Nijmegen	Nijmegen, Netherlands	Data contributor	
Emanuele	D'Amico		MD	Medical and Surgical Sciences, Universita di Foggia	Foggia, Italy	Data contributor	
Bianca	Weinstock-Guttman		MD	Department of Neurology, Jacobs MS center for treatment and research	Buffalo, New York, United States	Data contributor	
Vahid	Shaygannejad		MD	Isfahan University of Medical Sciences	Isfahan, Iran	Data contributor	
Yolanda	Blanco		MD	Center of Neuroimmunology, Service of Neurology, Hospital Clinic de Barcelona	Barcelona, Spain	Data contributor	
Jens	Kuhle		MD	Department of Neurology, University Hospital and University of Basel	Basel, Switzerland	Data contributor	
Francois	Grand'Maison		MD	Neuro Rive-Sud	Quebec, Canada	Data contributor	
Julie	Prevost		MD	CSSS Saint-Jérôme	Saint-Jerome, Canada	Data contributor	
Maria	Pia Amato		MD	Department NEUROFARBA, University of Florence	Florence, Italy	Data contributor	
Bassem	Yamout		MD	Neurology Institute and MS Center, Harley Street Medical Centre	Abu Dhabi, United Arab Emirates	Data contributor	
Samia	J. Khoury		MD	Nehme and Therese Tohme Multiple Sclerosis Center, American University of Beirut Medical Center	Beirut, Lebanon	Data contributor	
MSBase Foundation Centre Custodian				Institute of Neuroscience Buenos Aires	Buenos Aires, Argentina	Data contributor	
Murat	Terzi		MD	Medical Faculty, 19 Mayis University	Samsun, Turkey	Data contributor	
Celia	Oreja-Guevara		MD	Department of Neurology, Hospital Clinico San Carlos	Madrid, Spain	Data contributor	
Elisabetta	Cartechini		MD	Neurology Unit, AST Macerata	Macerata, Italy	Data contributor	
Guy	Laureys		MD	Department of Neurology, University Hospital Ghent	Ghent, Belgium	Data contributor	
Eduardo	Aguera-Morales		MD	Department of Medicine and Surgery, University of Córdoba	Córdoba, Spain	Data contributor	
Maria	Di Gregorio		MD	Neurology Unit, University Hospital San Giovanni di Dio e Ruggi d'Aragona	Salerno, Italy	Data contributor	
Ayse	Altintas		MD	Department of Neurology, School of Medicine and Koc University Research Center for Translational Medicine (KUTTAM), Koc University, School of Medicine	Istanbul, Turkey	Data contributor	
Recai	Turkoglu		MD	Department of Neurology, Haydarpasa Numune Training and Research Hospital	Istanbul, Turkey	Data contributor	
Marie	D'hooghe		MD	Department of Neurology, Nationaal MS Centrum	Melsbroek, Belgium	Data contributor	
Claudio	Solaro		MD	Neurology Unit, Galliera Hospital	Genova, Italy	Data contributor	
Aysun	Soysal		MD	Bakirkoy Education and Research Hospital for Psychiatric and Neurological Diseases	Istanbul, Turkey	Data contributor	
Jose	Antonio Cabrera-Gomez		MD	Centro Internacional de Restauracion Neurologica	Havana, Cuba	Data contributor	
Mario	Habek		MD, PhD	Department of Neurology, University Hospital Center Zagreb	Zagreb, Croatia	Data contributor	
Barbara	Willekens		MD, PhD	Department of Neurology, Antwerp University Hospital	Edegem (Antwerp), Belgium	Data contributor	
Masoud	Etemadifar		MD	Neurology Department, Dr. Etemadifar MS Institute, Isfahan University of Medical Sciences	Isfahan, Iran	Data contributor	
Maria	Edite Rio		MD	Centro Hospitalar Universitario de Sao Joao	Porto, Portugal	Data contributor	
Talal	Al-Harbi		MD	Neurology Department, King Fahad Specialist Hospital-Dammam	Dammam, Saudi Arabia	Data contributor	
Allan	G Kermode		MBBS, MD	Perron Institute for Neurological and Translational Science, Sir Charles Gairdner Hospital, QEIIMC, University of Western Australia	Western Australia, Australia	Data contributor	
Marzena	Fabis-Pedrini		PhD	Perron Institute for Neurological and Translational Science, Sir Charles Gairdner Hospital, QEIIMC, University of Western Australia	Western Australia, Australia	Data contributor	
William	M Carroll		MBBS, MD	Perron Institute for Neurological and Translational Science, The University of Western Australia	Perth, Australia	Data contributor	
Pamela	McCombe		MBBS	Department of Neurology, Royal Brisbane Hospital	Brisbane, Australia	Data contributor	
Jana	Houskova		MD	Department of Neurology, Hospital Ceske Budejovice	Ceske Budejovice, Czech Republic	Data contributor	
Eva	Recmanova		MD	Department of Neurology, Tomas Bata Hospital	Zlin, Czech Republic	Data contributor	
Ivana	Stetkarova		MD, PhD	Department of Neurology, Third Faculty of Medicine, Charles University in Prague and Hospital Kralovske Vinohrady	Prague, Czech Republic	Data contributor	
Magd	Zakaria		MD	Ain Shams University	Cairo, Egypt	Data contributor	
Nevin	Shalaby		MD	Cairo University, Kasr-Al-Ainy MS Clinic	Cairo, Egypt	Data contributor	
Tamara	Castillo-Triviño		MD	Hospital Universitario Donostia and IIS Biodonostia	San Sebastián, Spain	Data contributor	
Seyed	Mohammad Baghbanian			Neurology Department, Booalisina Hospital, Mazandaran University of Medical Sciences	Sari, Iran	Data contributor	

*First Name and Middle Initial(s)	*Last Name	*Suffix (eg, Jr, III)	Academic Degrees	Institution	Location (city, state/province, country)	Role or Contribution, eg, chair, principal investigator	Group (if more than 1 Group listed in the byline) and/or Subgroup (eg, Steering Committee)
Mihaela	Simu		MD	University of Medicine and Pharmacy Victor Babes	Timisoara, Romania	Data contributor	
Mark	Slee		BMBS, PhD	College of Medicine and Public Health, Flinders University	Adelaide, Australia	Data contributor	
Jennifer	Massey		MD	St Vincent's Hospital	Sydney, Australia	Data contributor	
Jiwon	Oh		MD	St. Michael's Hospital	Toronto, Canada	Data contributor	
Abdorreza	Naser Moghadasi			Multiple Sclerosis Research Center, Neuroscience Institute, Tehran University of Medical Sciences	Tehran, Iran	Data contributor	
Koen	de Gans		MD	Groene Hart Ziekenhuis	Gouda, Netherlands	Data contributor	
Abdullah	Al-Asmi		MD	College of Medicine & Health Sciences, Sultan Qaboos University	Al-Khodh, Oman	Data contributor	
Maria	Laura Saladino		MD	INEBA - Institute of Neuroscience Buenos Aires	Buenos Aires, Argentina	Data contributor	
Emmanuelle	Lapointe		MD	Medicine, Division of Neurology, Centre Hospitalier Universitaire de Sherbrooke	Quebec, Canada	Data contributor	
Claudio	Gobbi		MD	Ospedale Civico Lugano	Lugano, Switzerland	Data contributor	
Thor	Petersen		MD	Aarhus University Hospital	Arhus C, Denmark	Data contributor	
Ricardo	Fernandez Bolaños		MD	Hospital Universitario Virgen de Valme	Seville, Spain	Data contributor	
Stella	Hughes		MD	Royal Victoria Hospital	Belfast, United Kingdom	Data contributor	
Orla	Gray		MD	South Eastern HSC Trust	Belfast, United Kingdom	Data contributor	
Nikolaos	Grigoriadis		MD, PhD	Department of Neurology, AHEPA University Hospital	Thessaloniki, Greece	Data contributor	
Imre	Piroska		MD	Veszprém Megyei Csolnoky Ferenc Kórház zrt.	Veszprem, Hungary	Data contributor	
Canun	Yücesan			Neurology Department, Ankara University Ibni Sina Hospital	Ankara, Turkey	Data contributor	
Justin	Garber		MBBS, PhD	Department of Neurology, Westmead Hospital	Sydney, Australia	Data contributor	
Mike	Boggild		MBBS, PhD	Townsville Hospital	Townsville, Australia	Data contributor	
Danny	Decoo		MD	AZ Alma Ziekenhuis	Sijsele–Damme, Belgium	Data contributor	
Csilla	Rozsa		MD	Jahn Ferenc Teaching Hospital	Budapest, Hungary	Data contributor	
Tunde	Csepany		MD, PhD	Department of Neurology, Faculty of Medicine, University of Debrecen	Debrecen, Hungary	Data contributor	
Chris	McGuigan		MD	St Vincent's University Hospital	Dublin, Ireland	Data contributor	
Mehmet	Fatih Yetkin		MD	Neurology Department, Erciyes University, Neurology	Kayseri, Turkey	Data contributor	
Norma	Deri		MD	Neurology Department, Hospital Fernandez	Capital Federal, Argentina	Data contributor	
Edgardo	Cristiano		MD	Centro de Esclerosis Múltiple de Buenos Aires (CEMBA)	Buenos Aires, Argentina	Data contributor	
Carlos	Vrech		MD	Sanatorio Allende	Córdoba, Argentina	Data contributor	
Neil	Shuey		MBBS	St Vincent's Hospital, Fitzroy	Melbourne, Australia	Data contributor	
Todd	A. Hardy		MBBS, PhD	Department of Neurology, Concord Repatriation General Hospital	Sydney, Australia	Data contributor	
Steve	Vucic		MBBS, PhD	Concord Repatriation General Hospital	Sydney, Australia	Data contributor	
Stephen	Reddel		MBBS, PhD	Concord Repatriation General Hospital	Sydney, Australia	Data contributor	
Sudarshini	Ramanathan		MBBS, PhD	University of Sydney and Department of Neurology, Concord Clinical School, Concord Hospital	Sydney, Australia	Data contributor	
Deborah	Field			Lyell McEwin Hospital, Neurology, Elizabeth Vale	South Australia, Australia	Data contributor	
Bart	Van Wijmeersch		MD, PhD	University MS Centre, Hasselt-Pelt and Noorderhart Hospitals, Rehabilitation & MS	Pelt, Belgium	Data contributor	
Melissa	Cambron		MD, PhD	Neurology Department, Az Sint-Jan Brugge	Bruges, Belgium	Data contributor	
Simón	Cárdenas-Robledo			Hospital Universitario Nacional de Colombia Bogotá and Centro de Esclerosis Múltiple (CEMHUN), Departamento de Neurología	Bogotá, Colombia	Data contributor	
Jose	Luis Sanchez-Menoyo		MD	Department of Neurology, Galdakao-Usansolo University Hospital, Osakidetza-Basque Health Service	Galdakao, Spain	Data contributor	
Krisztina	Kovacs		MD	Péterfy Sandor Hospital	Budapest, Hungary	Data contributor	
Attila	Sas		MD	Department of Neurology and Stroke, BAZ County Hospital	Miskolc, Hungary	Data contributor	
Eniko	Dobos		MD	Szent Imre Hospital	Budapest, Hungary	Data contributor	
Bhim	Singhal		MD	Bombay Hospital Institute of Medical Sciences	Mumbai, India	Data contributor	
Norio	Chihara			Division of Neurology, Kobe University Graduate School of Medicine	Kobe, Japan	Data contributor	
Marija	Cauchi		MD	Neurosciences Department, Mater Dei Hospital	Birkirkara, Malta	Data contributor	
Deborah	Mason		MBChB	Christchurch Hospital	Christchurch, New Zealand	Data contributor	
Jabir	Alkhaboori		MD	Department of Neurology, Royal Hospital	Muscat, Oman	Data contributor	
Ilya	Kister		MD	New York University Langone Medical Center	New York, United States	Data contributor	
