# Supplementary material for: Pregnancy-Related Disease Outcomes in Women With Moderate to Severe Multiple Sclerosis Disability
Source: JAMA Netw Open. 2025 Sep 15;8(9):e2531581. doi: 10.1001/jamanetworkopen.2025.31581 (PMC12439057; doi:10.1001/jamanetworkopen.2025.31581)
Supplement: Supplement 3. — Data Sharing Statement [file jamanetwopen-e2531581-s003.pdf]

Data Sharing Statement

Shipley. Pregnancy-Related Disease Outcomes in Women With Moderate to Severe Multiple Sclerosis Disability. *JAMA Netw Open*. Published September 15, 2025. doi:10.1001/jamanetworkopen.2025.31581

Data
Data available: Yes
Data types: Deidentified participant data
How to access data: vilija.jokubaitis@monash.edu
When available: With publication

Supporting Documents
Document types: None

Additional Information
Who can access the data: MSBase is a data processor and warehouses data from individual Principal Investigators who agree to share datasets on a project-by-project basis. Data access to qualified researchers can be granted at the discretion of each Principal Investigator. Contact the corresponding author for further details.
Types of analyses: Academic purposes only.
Mechanisms of data availability: After approval of a proposal, with a signed data access agreement, and with permission from each individual data controller.
